# Oligodendrocyte Slc48a1 (Hrg1) encodes a functional heme transporter required for myelin integrity

**DOI:** 10.1002/glia.24641

**Published:** 2024-11-06

**Authors:** John H. Stockley, Adrien M. Vaquie, Zhaoyang Xu, Theresa Bartels, Gregory D. Jordan, Staffan Holmqvist, Simon Gunter, Guy Lam, Daniel Yamamoto, Rini H. Pek, Ian G. Chambers, Andrew S. Rock, Myfanwy Hill, Chao Zhao, Scott Dillon, Robin J. M. Franklin, Rosemary O'Connor, David M. Bodine, Iqbal Hamza, David H. Rowitch

**Affiliations:** ^1^ Wellcome—MRC Cambridge Stem Cell Institute, University of Cambridge Cambridge UK; ^2^ Department of Paediatrics Biomedical Campus, University of Cambridge Cambridge UK; ^3^ Department of Pediatrics Center for Blood Oxygen Transport and Hemostasis, University of Maryland School of Medicine Baltimore Maryland USA; ^4^ Department of Animal and Avian Sciences University of Maryland Maryland USA; ^5^ Department of Clinical Neurosciences Biomedical Campus, University of Cambridge Cambridge UK; ^6^ School of Biochemistry and Cell Biology, University College Cork Cork Ireland; ^7^ Haematopoiesis Section, Genetics and Molecular Biology Branch, National Human Genome Research Institute Bethesda Maryland USA

**Keywords:** axon, heme, heme oxygenase, Hrg1, iron deficiency, myelin, myelin associated glycoprotein (Mag), myelin basic protein (Mbp), neurodegeneration, oligodendrocyte

## Abstract

Oligodendrocytes (OLs) of the central nervous system require iron for proteolipid biosynthesis during the myelination process. Although most heme is found complexed to hemoglobin in red blood cells, surprisingly, we found that *Slc48a1*, encoding the heme transporter Hrg1, is expressed at higher levels in OLs than any other cell type in rodent and humans. We confirmed in situ that *Hrg1* is expressed in OLs but not their precursors (OPCs) and found that Hrg1 proteins in CNS white matter co‐localized within myelin sheaths. In older *Hrg1* null mutant mice we observed reduced expression of myelin associated glycoprotein (Mag) and ultrastructural myelin defects reminiscent of *Mag*‐null animals, suggesting myelin adhesion deficiency. Further, we confirmed reduced myelin iron levels in *Hrg1* null animals in vivo, and show that OLs in vitro can directly import both the fluorescent heme analogue ZnMP and heme itself, which rescued iron deficiency induced inhibition of OL differentiation in a heme‐oxidase‐dependent manner. Together these findings indicate OL *Hrg1* encodes a functional heme transporter required for myelin integrity.

## Main Points

- Hrg1 is a heme transporter in myelin.
- Heme is an auxiliary source of iron for oligodendrocytes and required for Mag expression.
- Hrg1 KO mice have increased periaxonal space due to decreased levels of Mag.

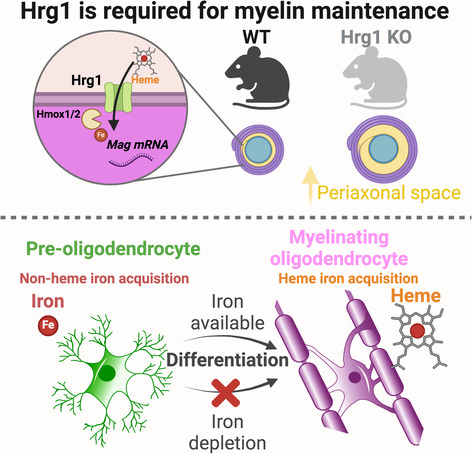

## 1 INTRODUCTION

Myelin, the insulating sheath that surrounds neuronal axons, is produced by oligodendrocytes (OLs) in the central nervous system (CNS). It is an evolutionary innovation, which first appears in vertebrates above jawless fishes, enabling more rapid transmission of nerve impulses at reduced axon diameter (Stadelmann et al., 2019), both critical to house a larger number of neurons and hence greater brain complexity (Freeman and Rowitch, 2013). However, to produce myelin segments for perhaps hundreds of axons, oligodendrocyte precursors (OPCs) undergo a ~6500‐fold increase in cell surface area during differentiation and myelination (Chrast et al., 2011), and as such requirements for iron‐dependent biosynthetic processes are very high. This explains in part the observation that iron deficiency during development significantly impairs CNS myelination (Yu et al., 1986; Badaracco et al., 2010; Isasi et al., 2022) and neurocognitive abilities in animal models and humans (Lozoff et al., 2006; Todorich et al., 2009).

Cellular iron acquisition can occur by several mechanisms. Indeed, the elemental iron transporter Dmt1 (*Slc11a2*) and the transferrin receptor (Tfrc) are essential for OPC maturation into mature OLs (Connor et al., 1990; Taylor and Morgan, 1990; Todorich et al., 2009; Cheli et al., 2018; Möller et al., 2019; Cheli et al., 2023). Dmt1 transports iron from the extracellular environment across the plasma membrane but also from the lumen of endosomes and lysosomes after Tfrc mediated internalization of iron (Cheli et al., 2018, 2023). Deletion of the Tfrc receptor in OPCs impairs myelination but Tfrc ablation in mature OLs after myelination is established has no effect (Cheli et al., 2023). Heme is a major carrier for iron provision in the body, and an essential co‐factor for oxygen transport in hemoglobin, electron transport in mitochondria and enzymatic activities of peroxidases and monooxygenases (Chambers et al., 2021). *Hrg1*, encoded by *Slc48a1*, is an ancient evolutionarily conserved heme transporter required for survival in heme auxotrophs and iron recycling during erythrophagocytosis in vertebrates (Rajagopal et al., 2008; Zhang et al., 2018; White et al. 2013). For example, *Haemonchus contortus* (aka Barber's pole worm) lack the ability to synthesize heme and acquire it via the gut, which can be demonstrated by the uptake of the fluorescent heme analogue ZnMP after ingestion but dependent upon the presence of Hrg1 (Yuan et al., 2012; Yang et al., 2023).

While the brain tissues are not regarded as containing particularly high levels of heme, high levels of iron have been reported in OLs and their myelin sheaths (Connor et al., 1990; Meguro et al., 2007; Todorich et al., 2009; Badaracco et al., 2010; Möller et al., 2019). We therefore investigated iron metabolism by single‐cell transcriptomics and found, surprisingly, that *Hrg1* is expressed at its highest levels in mature myelinating OLs across all tissues including hematopoietic lineages. We confirmed *Hrg1* expression in situ in OLs and that Hrg1 proteins are contained within myelin sheaths. Loss of *Hrg1* function in vivo resulted in significant and heterogeneous forms of dysmyelination at the ultrastructural level, associated with lower levels of myelin associated glycoprotein (Mag). Finally, we found that heme could directly rescue OPC differentiation into OLs in the setting of iron depletion. Our findings indicate heme import comprises an accessory pathway for iron acquisition and plays an essential role in maintenance of myelin integrity in adult mice.

## 2 METHODS

### 2.1 Resources

A detailed list of key resources used in this study are given in Table S1.

### 2.2 Animals

Mice in all studies were of mixed gender. All animal protocols were approved by the Institutional Animal Care and Use Committee at the University of Maryland, College Park (IACUC Animal Study Protocol R‐NOV‐18–61). Animals were housed under standard 12‐hour light/dark cycle conditions and were fed ad libitum. *Hrg1* mutant mice have been generated as described previously (Pek et al., 2019). Rats were housed at the University of Cambridge, under standard laboratory conditions on a 12‐h light/dark cycle with constant access to food and water and studies were conducted under the Animals (Scientific Procedures) Act 1986 Amendment Regulations 2012 following ethical review by the University of Cambridge Animal Welfare and Ethical Review Body.

### 2.3 Tissue processing and immunofluorescence

Unless otherwise stated all chemicals were sourced from Sigma‐Aldrich UK (Merck, UK). Mice were sacrificed by cardiac perfusion using Dulbecco's phosphate‐buffered saline (DPBS) (Thermo Fisher Scientific, USA) under anesthesia (10% ketamine, 8% xylazine mix) and perfused with 4% w/v paraformaldehyde (PFA) in DPBS for fixation. Specimens were placed in 50 mL centrifuge tubes and shipped to the University of Cambridge for analysis. Mouse specimens shipped from University of Maryland were dissected and brains removed. Brains were further fixed overnight in 4% PFA and cryoprotected in 20% (w/v) sucrose for 48 h. Tissue was embedded in blocks of OCT matrix (Fisher Scientific, UK), cryogenically frozen and cryosectioned (16 μm thick) using a Leica CM3050S cryostat (Leica Microsystems, UK) onto Superfrost Plus microscope slides (Fisher Scientific, UK), air dried and frozen immediately at −80°C until use. Frozen tissue sections were allowed to equilibrate to room temperature (RT) before being dried in an oven at 65°C for 30 min. Sections were then hydrated in PBS pH 7.4 at RT (room temperature) for 5 min. Antigen retrieval (AR) was performed using citrate buffer pH 6.0 from a 10X stock, for 10 min at 80°C using a water bath. However, AR was avoided for Hrg1 immunostaining. Sections were incubated in either 10% (v/v) normal donkey serum in PBS with 0.02% (v/v) Triton X‐100 or 3% (w/v) BSA with 0.003% (v/v) Triton X‐100 for 1 h at RT on a hula‐mixer (Thermo Fisher Scientific, UK). Sections were encircled with a wax Pap pen (Abcam, UK) and incubated in primary antibodies at the following dilutions (Mbp, 1:100; Mog, 1:200; Sox10, 1:100; rabbit anti‐Hrg1 from Rosemary O'Connor University College Cork, 1:100; rabbit anti‐Hrg1 from Iqbal Hamza University of Maryland, 1:200, NF‐H, 1:500; Mag, 1:100; Plp1, 1:500; Aspa, 1:100) overnight at 2–8°C. Sections were washed for 1 × 15 min and 3 × 5 min in PBS and incubated with compatible secondary Alexa Fluor antibodies (Thermo Fisher Scientific, UK) 1:500 dilution for 1 h at RT with Hoechst 33342 (1:5000 dilution) and washed for 1 × 15 min and 3 × 5 min in PBS. Excess PBS was removed and slides were mounted in ProLong Gold Antifade (Thermo Fisher Scientific, UK), cover‐slipped (thickness = 1.5) (Fisher Scientific, UK) and allowed to dry. Slides were imaged using a Zeiss Apotome 2 or Nikon 90i for epifluorescent imaging. For confocal imaging either an Operetta CLS (PerkinElmer, UK) with 40X water immersion objective or a Leica SP5 (Leica Microsystems, UK) with 63X oil immersion objective. For stimulated emission depletion (STED) microscopy, standard immunostaining protocols were used with the exception of 10% (v/v) normal goat serum in replacement of donkey serum, incubation of primary antibodies for 18 h, omission of Hoechst and incubating with STED compatible secondary antibodies (Table S1) (Abberior Instruments GmBH, DE) for 2 h at RT and doubling of the washing steps at all stages. STED imaging was conducted using a Zeiss Axioscope with STEDYcon (Abberior Instruments GmBH, DE) using a 100X oil immersion objective.

### 2.4 Single molecules fluorescent in situ hybridization (smFISH)

Mouse smFISH was performed using the RNAScope LS Multiplex Assay (Biotechne, UK) as previously described (Bayraktar et al., 2020). RNAScope probes are hybridized to mouse tissue on microscope slides followed by immunofluorescence for NeuN and nuclei staining with DAPI (0.2 μg/mL) using an automated Leica Bond RX (Leica Microsystems, UK). Tissue slides were mounted in ProLong Gold Antifade (Thermo Fisher Scientific, UK), cover‐slipped (thickness = 1.5H) (Paul Marienfeld GmbH & Co. KG, DE). Tissue sections were imaged on an automated spinning disk confocal microscope Operetta CLS (PerkinElmer, UK) using 5X and 20X air objectives as well as a 40X water immersion objective.

### 2.5 Transmission electron microscopy

Tissue samples for electron microscopy were prepared according to Karlsson and Schultz (1965). Mice were transcardially perfused with 4% PFA, 0.25% glutaraldehyde and 0.5% NaCl in phosphate buffer pH 7.4 and remained in fixative for a further 3 days. Specimens were placed in 50 mL centrifuge tubes and shipped to Cambridge for further processing. Optic nerves were carefully dissected and fixed further by immersing them in a solution containing 1% aqueous osmium tetroxide and 1.5% potassium ferricyanide overnight at 4 °C. After thorough washing in deionized water (dH_2_O), the samples were en‐bloc‐stained in a 3% aqueous uranyl acetate solution (Agar Scientific, UK) for 24 h at 4°C. Subsequently, the samples underwent dehydration through an ethanol series, followed by infiltration with a 1:1 mixture of propylene oxide and resin. Blocks of fresh resin were then polymerized at 60°C for 48 h. Ultrathin sections, approximately 60 nm in thickness, were cut from the resin blocks using an EM UC7 ultramicrotome (Leica Microsystems, UK) and placed on copper grids coated with carbon and formvar (Agar Scientific, UK). The grids were post‐stained with uranyl acetate and lead citrate before being imaged with a HT7800 transmission electron microscope (Hitachi High Technologies, UK) operating at 100 kV.

### 2.6 Non‐heme iron staining

Tissue non‐heme iron was stained as previously described (Schirmer et al., 2019) with minor modifications based on work by Meguro et al. (2007). Sections of fixed wild type and Hrg1 mutants were studied in parallel. Frozen tissue sections were allowed to warm to RT and dried for 15 min in a laminar‐flow hood. Endogenous peroxidase activity was quenched by immersion in a solution of 0.3% H_2_O_2_ (v/v) in methanol for 20 min and washed three times in dH_2_O. Sections were then placed in a fresh solution of 1% (w/v) potassium ferricyanide, 0.1% Triton‐X 100 (v/v), 5% (w/v) polyvinylpyrrolidone (PVP) with 1% (v/v) HCl overnight on a HulaMixer (Thermo Fisher Scientific, UK), followed by three washes in dH_2_O. Sections bathed in 0.01 M NaN_3_, 0.3% H_2_O_2_ and methanol for 60 min on a HulaMixer, followed by three washes in PBS. Iron staining was intensified using DAB (10% v/v) solution from Pierce DAB substrate kit (Thermo Fisher) in PBS with 0.005% H_2_O_2_ (v/v) until optimal intensity in control tissues was reached between 30 and 90 min. Negative controls were included in all test by omitting potassium ferricyanide resulting in no Prussian blue and DAB precipitation.

### 2.7 Image analysis

Unless otherwise stated all images were processed and analyzed using Fiji v 2.9.0. For 3D rendering the Fiji plugin Volume J1.8 was used with raytrace rendering algorithm, classifier threshold and deviation of 192 and 1 respectively with trilinear interpolation. All images acquired on the Operetta CLS were analyzed using Harmony software (Perkin Elmer, UK) or OMERO.web 5.22.1 (University of Dundee, UK). For smFISH experiments, single cells were segmented as described (Bayraktar et al., 2020) using either *Ermn* (OLs), *Pdgfra* (OPCs) or *Syt1* (neurons) NeuN (neurons) as cell type markers, and the number of *Hrg1* mRNA spots was calculated per single cell. All images acquired on Operetta CLS were analyzed using Harmony (PerkinElmer, UK). Fluorescent intensity and DAB staining intensities were measured using either Fiji v 2.9.0 or OMERO.web 5.22.1 (University of Dundee, UK). Transmission electron microscopy images were processed using HT7800 TEM operating software v.01.21 (Hitachi High Technologies, UK) and analyzed using Fiji v 2.9.0 software and MyelTracer (Kaiser et al., 2021).

### 2.8 Myelin fractionation

Myelin fractions were prepared as previously described (Schirmer et al., 2019). Unless specified, all buffers were prepared in DEPC‐treated water, and procedures were conducted at 2–8 °C. Adult Wistar rats (3 months old) were terminally anesthetized and intracardially perfused with saline and heparin (5 IU/mL), brains rapidly dissected, and olfactory bulbs removed. The remaining brain was kept on ice, homogenized in 12 mL of 0.32 M sucrose (DEPC‐treated water) with HALT protease inhibitor cocktail (Thermo Fisher Scientific, UK). After homogenization, 1.5 mL of the homogenate was retained, and 6 mL was loaded on top of 6 mL of 0.85 M sucrose (DEPC‐treated water) with HALT protease inhibitors. Centrifugation at 75,000 g for 35 min at 4 °C (Beckman SW40Ti rotor) yielded the pellet (P1) and the 0.85 M–0.32 M sucrose interface, collected as total myelin (T.M.). Thirty percent of the T.M. fraction was retained, and the remainder was resuspended in water and centrifuged at 75,000 g for 15 min at 4°C. The resulting pellet underwent two rounds of osmotic shock, resuspended in 6 mL of 0.32 M sucrose, and overlaid on a bed of 0.85 M sucrose. Centrifugation at 75,000 g for 35 min at 4°C collected the compact myelin (C.M.) fraction from the 0.32 M to 0.85 M sucrose interface. The C.M. fraction was washed, centrifuged, and resuspended in tris‐buffered saline (TBS) pH 7.4 to a final volume of 400 μL. All fractions were homogenized with 10 strokes using a glass dounce, and protein content was determined using a fluorescent Qubit protein assay (Thermo Fisher Scientific, UK) on a Spectromax microplate reader (Molecular Devices, UK) at 470 nm excitation and 580 nm emission.

### 2.9 Western blotting

Mouse brain tissue from Hrg1 mutants and littermate controls were homogenized using glass dounce in 10 volumes of PBS with 0.32 M sucrose and HALT protease inhibitors. Samples were centrifuged at 300 g for 5 min at 4°C to remove unhomogenized debris and the supernatants retained and homogenized further using 10 strokes with a glass dounce. Protein concentrations were determined using Pierce BCA Protein assay kit (Thermo Fisher Scientific, UK) and Qubit protein assay as described above. A total of 15 μg protein from mouse brain lysates or myelin fractions were separated on either 4%–12% Bis‐Tris NuPAGE gels (Thermo Fisher Scientific, UK) or Novex 10%–20% Tris‐Tricine gels (Thermo Fisher Scientific, UK) according to the manufacturer's instructions after heat induced denaturation. Voltages of 90 V for 15 min followed by 120 V were applied, until the dye front reach the end of the gel. Proteins were transferred to PVDF membranes (Immobilon‐FL 0.45 μ, Merck UK) using Bolt Transfer buffer (Thermo Fisher Scientific, UK) at 15 V for 90 min at 2–8°C. Membranes were blocked for 1 h in blocking buffer consisting 50% (v/v) Intercept Blocking Buffer (LI‐COR Biosciences, UK) in TBS with 0.1% (v/v) Tween‐20 (Merck, UK) (TBS‐T). Primary antibodies were diluted in blocking buffer and incubated overnight at 2–8°C with mixing. Membranes were washed 1 × 15 min and 3 × 5 min in TBS‐T and incubated with respective Li‐COR secondary antibodies (1:5000) for 1 h at RT. Membranes were washed again 1 × 15 and 3 × 5 min in TBS‐T. Proteins were detected using a Li‐COR Odyssey (LI‐COR Biosciences, UK) and analyzed using ImageStudio v5.2.5 (LI‐COR Biosciences, UK). For protein staining, freshly run Tris‐Tricine gels were treated with Pierce Silver Stain Kit (Thermo Fisher Scientific, UK) according to manufactures instructions and imaged using a flatbed scanner.

### 2.10 Cholesterol assay

Lipids were purified from myelin fractions using Folch method (Folch et al., 1957). A total of 50 μg protein was brought to 50 μL volume with TBS in microfuge tube. To this 300 μL of chloroform:methanol (2:1) was added and vigorously mixed for 10 min at RT. A total of 75 μL dH_2_O was added, vortexed to mix, followed by centrifugation at 2000 g for 5 min. The aqueous phase was removed and organic phase was kept. The microfuge tubes were placed in a vacuum centrifuge (Eppendorf, UK) for 5 h and evaporated to dryness. A total of 25 μL methanol was added to dissolve lipids and vortexed to mix. Amplex‐Red cholesterol assay (Thermo Fisher Scientific, UK) was performed according to manufacturer's instructions. Briefly, 5 μL of purified lipids was diluted in 50 μL of reaction buffer in a black walled fluorescent microwell plate (Fisher Scientific, UK) and 50 μL of Amplex‐Red working reagent with HRP, cholesterol esterase, cholesterol oxidase, and Amplex‐Red was added, mixed thoroughly and incubated at 37°C for 30 min. Reaction was measured with 550 nm excitation and 590 nm emission on a Spectromax microplate reader (Molecular Devices, UK) and analyzed against a cholesterol standard curve.

### 2.11 Primary rat oligodendrocytes

The following steps were modified from (Neumann et al., 2019). Euthanized and decapitated Wistar rat neonates of post‐natal days 4–7 (P4–P7) were purchased from Charles River Laboratories, UK and shipped same day at 2–8°C in Hibernate‐A medium (Thermo Fisher Scientific, UK). Brains were harvested into Hibernate‐A (Thermo Fisher Scientific, UK), subsequently, olfactory bulbs were removed, and the brains were mechanically minced into small pieces (approx. 1 mm^2^) using a sterile scalpel. Tissue pieces were resuspended in HBSS (Thermo Fisher Scientific, UK) and pelleted at 100 g for 3 min at RT. The resulting tissue pellet was then resuspended in dissociation medium consisting of 34 U/mL papain (Worthington Labs, via Lorne UK) and 20 μg/mL DNase‐1 in Hibernate A for 30 min at 37°C on a horizontal shaker (50 r.p.m.). Brain homogenate was collected by centrifugation for 5 min at 300 g at RT and resuspended in a neutralizing solution comprising 1X B27 (Thermo Fisher Scientific, UK), 2 mM sodium pyruvate in Hibernate A. Tissues were then triturated sequentially using a 10‐ml pipet, 5 mL pipet, and a sterile fire‐polished Pasteur pipet to generate a single‐cell suspension. The resulting cell suspension was filtered through a 70 μm cell strainer (Greiner, UK) into a Percoll solution (Cytiva, UK) to achieve a final Percoll concentration of 22.5%. The mixture was subsequently centrifuged at 800 g for 20 min with no break in centrifugation deceleration. The supernatant was carefully removed, and the cell pellet was washed once with HBSS. To eliminate red blood cells from the cell suspension, the cells were resuspended in 1 mL of Red Blood Lysis Buffer (Merck, UK) for 1 min, followed by washing with a large volume of HBSS. The cell suspension was then resuspended in 500 μL of washing buffer (WB) consisting of 2 mM EDTA, 2 mM sodium pyruvate, 0.5% bovine serum albumin (BSA) in PBS pH 7.4 supplemented with 25 μg/mL insulin (Merck, UK). Subsequently, 1 μL of mouse monoclonal antibody against A2B5 (Merck, UK) was added to the suspension and the cells were incubated for 30 min at 2–8°C with gentle shaking. Following incubation, cells were washed with WB, harvested by centrifugation (300 g, 5 min, RT), resuspended in 80 μL of WB supplemented with 25 μg/mL insulin and 20 μL of rat anti‐mouse IgM antibody (Miltenyi Biotec, UK), and incubated for 15 min at 4°C. After incubation, the cells were washed with 8 mL of WB, resuspended in 2 mL of WB supplemented with insulin, and loaded into a MACS LS column (Miltenyi Biotec, UK). A2B5 positive cells were isolated in a MACS Multistrand (Miltenyi Biotec, UK) according to the manufacturer's instructions and resuspended in OPC media (Base media: DMEM/F12 (Thermo Fisher Scientific, 11039‐021), 2 mM Sodium pyruvate, 60 μg/mL N‐acetyl Cysteine, 25 μg/mL Insulin, and 1X SATO (SATO 100X: 1.61 mg/mL putrecine, 4 μg/mL sodium selenite, 60 μg/mL Progesterone, 33 mg/mL BSA, 5 mg/mL Apo‐Transferrin in DMEM/F12) with 20 ng/mL PDGF‐aa (Peprotech, 100‐13A) + 20 ng/mL bFGF (Peprotech, 100‐18B) for 2–6 days in a humidified incubator at 37°C, 5% CO_2_, 90% N_2_, or 5% O_2_ for low oxygen and at 37°C, 5% CO_2_ and ambient oxygen for high oxygen experiments. At the desired confluency (day 2 or 3), the media was changed to OL media (Base media + 40 ng/mL T3) to induce differentiation into OLs. Drug treatments were done using OL media supplemented with 100 μM Deferoxamine (DFO) (Merck, UK), 1 μM Hemin (Merck, UK) or 5 μM SnPP (Santa Cruz Biotechnology, USA). Cells were washed twice with PBS and fixed for 15 min in 4% PFA or mRNAs were collected in Tri‐Reagent solution (Thermofisher Scientific, UK). Immunostaining was performed as per tissue sections with blocking, primary antibody incubation, washing steps and secondary antibody incubation and imaged using a Leica DMI6000 (Leica Microsystems, UK) to acquire wide‐field images.

### 2.12 Primary mouse mixed glia

Cultures were prepared based on methods established elsewhere with modifications (Jia et al., 2018). Euthanized and decapitated C57 mouse pups of post‐natal days 1–2 (P1–P2) were purchased from Charles River Laboratories, UK, and shipped same day at 2–8°C in DMEM/F12 (Thermo Fisher Scientific, UK). Upon arrival, in a sterile laminar‐flow hood, CNS cortical hemispheres were rapidly dissected, cross chopped in cold Hibernate and debris removed through a Percoll gradient as for OPC cultures. Cells were suspended in base media with the inclusion of HB‐EGF at 5 ng/mL and plated onto poly‐d‐lysine coated surfaces, 6 cortices per 75 cm^2^. Complete media was replaced after 1.5 h to remove debris and un‐associated cells. Half of the media was changed twice up to the fourth day in vitro. HB‐EGF was removed and replaced with 40 ng/mL T3 in base media to promote OPC differentiation into mature OLs and retain astrocyte and microglia cells. Complete media was changed every other day for a further 10 days.

### 2.13 ZnMP uptake assay

Primary rat oligodendrocytes and primary mouse mixed glia were treated with Zn(II) Mesoporphyrin IX (ZnMP) (Santa Cruz Biotechnology, USA) using methods previously described with minor modifications (Rajagopal et al., 2008; O'Callaghan et al., 2010). Rat oligodendrocytes and mouse mixed glia at days 6 and 14, respectively, were washed with uptake medium made of 25 mM HEPES in HBSS supplemented with 2.5 μM BSA. A 10 mM ZnMP stock was added to uptake medium to a final of 5 μM and added to cells for 30 min. Cells were placed on ice and washed three times with ice cold uptake medium, followed by washing with PBS before fixing with 4% PFA dissolved in PBS for 10 min. Cells were stained with or without antibodies and the autofluorescence of ZnMP detected using an Operetta CLS or Leica SP5 confocal microscopes as above. For quadruple staining experiments excitation (ex.) and emission (em) filter sets for DAPI (ex. 355–385 nm, em 430–500 nm), Alexa‐488 (460–490 nm, em. 500–550 nm), Texas Red (ex. 530–560 nm, em 570–650 nm) and Alexa‐647 (ex. 615–645 nm, em 655–760 nm) filter sets on the Operetta CLS were chosen.

### 2.14 Quantitative real‐time PCR

Cells were washed with ice cold PBS and lysed with Tri‐Reagent solution (Thermo Fisher Scientific, UK). RNA was purified using Direct‐zol RNA MicroPrep (Cambridge Bioscience, UK) and cDNA generated using SuperScript 3 first‐Strand cDNA Kit (Thermo Fisher Scientific, UK) according to manufacturer's instructions. Quantitative real‐time PCR (qRT‐PCR) analyses were performed with a QuantStudio 12 K Flex Real time PCR system (Applied biosystems) using PowerUp SyBr Green Master Mix (Applied biosystems, A25741) according to manufacturer's instruction. Gene expression levels were normalized to β‐actin gene expression and analyzed using GraphPad Prism 9 software (GraphPad Software/Dotmatics, USA).

### 2.15 MTT and cell viability assays

Cell viability assays were performed on primary OPC and OL cultures using the manufacturers recommendations. For MTT assay (Abcam, UK), OPCs, and OL cultures were grown in flat bottom 96 well plates (Greiner, UK) at 5% O_2_, and treated with increasing concentrations hemin for 24 h. The media was removed from each well and 100 μL of MTT reagent in cell culture media 50% (v/v) was added and cells placed back in the incubator for 1–2 h. 150 μL of MTT solvent was added and mixed well and conversion of MTT to formazan was measured using a SPECTROstar Nano (BMG Labtech, UK) measuring absorbance at 590 nm. Data were analyzed with MARS software (BMG Labtech, UK) and Graphpad Prism 9 (GraphPad Software/ Dotmatics, USA).

For Live/Dead Assay (Thermo Fisher Scientific, UK), cells were treated with 20 μM Hemin from a 10 mM stock dissolved in 40 mM NaOH, or 87 μM Ferric citrate (20 μM Fe^3+^) for 24 h. Calcein AM and ethidium homodimer (Et. H.) were dissolved to concentrations of 1 μM and 0.5 μM, respectively, in cell culture media. Complete media was replaced with assay mixture (Calcein AM and Et. H.) and cells incubated for 20 min. Cells were then washed three times with PBS, fixed with 4% PFA for 10 min and counterstained with Hoechst 33342 (1:5000) for 1 h in PBS. Cells were imaged using an Operetta CLS (PerkinElmer, UK) and analyzed using Harmony software (PerkinElmer, UK).

### 2.16 Bioinformatics

Publicly available single‐cell RNAseq data sets for mouse CNS (Zeisel et al., 2018), mouse CNS development (Rosenberg et al., 2018), and human (Karlsson et al., 2021) was used as well as the Human Protein Atlas (https://www.proteinatlas.org/ENSG00000211584-SLC48A1/single+cell+type). Single‐cell sequence analysis was done using Scanpy (version 1.8.1) to generate the matrix plot. Specifically, for the investigation of iron binding pathways, we calculated the mean expression of all genes within the designated gene list. To ensure consistency and comparability across samples, gene expression values were normalized to 1^6 and subsequently logged. For the visualization of cellular heterogeneity, UMAP plots were generated using Scanpy as well. Dimension reduction focused on the top 2048 high variable genes identified by scanpy with “seurat‐v3” algorithm and using the DESC algorithm (version 2.1.1) for further processing. Trajectory analysis was carried out using the PAGA pipeline developed by (Wolf et al., 2019), following a standardized procedure. This comprehensive methodological approach allowed us to unravel the intricacies of single‐cell gene expression patterns and provided insights into the dynamic processes associated with iron binding pathways.

### 2.17 Statistical analyses

Numbers of experiments are indicated on bar graphs as individual data points, data shown as mean ± standard error of the mean (S.E.M.), and assumed to follow normal distribution. All data were analyzed using GraphPad Prism version 9.0.0 for Mac (GraphPad Software/Dotmatics, USA). EC_50_ values were calculated using a non‐linear regression and constraining *F* = 50. P values from Student's two tailed unequal variance t‐tests (Welch's correction) and *p* values <.05 were considered significant.

### 2.18 Illustrations

All art work was created using Adobe Illustrator CC 21.1.0 (Adobe Systems, USA) or BioRender (www.biorender.com) under the terms of an academic license.

## 3 RESULTS

### 3.1 *Hrg1* expression is enriched in mature myelinating OLs

**FIGURE 1:**
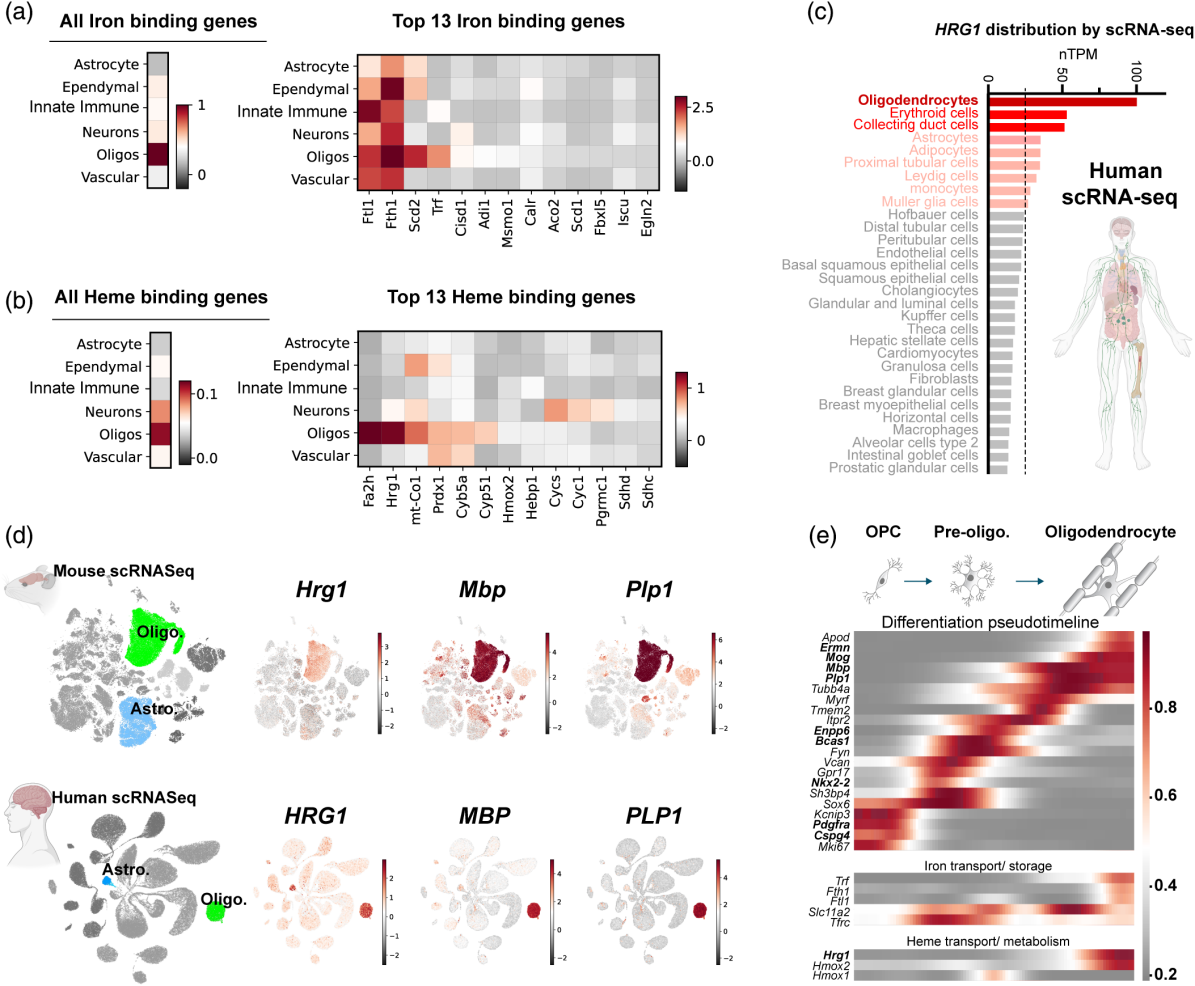
*Hrg1* (*Slc48a1*) expression is enriched in mature myelinating oligodendrocytes. (a and b) Heat map displaying the expression of all iron and heme binding genes (columns) across six central nervous system (CNS) cell types (rows), identified through gene ontology terms GO:0005506 (iron ion binding) and GO:0020037 (heme binding genes). The top 13 highest expressed transcripts are highlighted. Expression levels were normalized and shown in log scale. See also Figure S1. (c) Single‐cell RNAseq expression profile of human *HRG1* across human cells obtained from the Human Protein Atlas (www.proteinatlas.org/ENSG00000211584‐SLC48A1/single+cell+type). (d) Uniform Manifold Approximation and Projection (UMAP) depicting the single RNA sequencing from mouse and human CNS cell types for *Hrg1*/*HRG1* (mouse/human orthologues) alongside mature oligodendrocyte markers *Mbp*/*MBP* and *Plp1*/*PLP1*. (e) Pseudo‐timeline illustrating the expression dynamics of selected transcripts from SPLiT‐Seq (Rosenberg et al., 2018) data of oligodendroglial lineage cells from P2 and P11 CNS, highlighting the differentiation of oligodendrocyte precursor cells (OPC) into mature oligodendrocytes (OLs). Heme and iron metabolic transcripts are included for comparison.

Heme and iron are essential for life, but present a biological paradox as excessive levels of iron or heme are toxic to cells (Rouault, 2013; Chambers et al., 2021). Significant efforts have addressed iron cycling in the CNS, however in comparison, heme metabolism is poorly understood. Iron and heme binding genes constitute about 2% of the genome (Andreini et al., 2018). To gain insight into OL iron metabolism, we analyzed iron pathway expression data from previous studies (Pek et al., 2019) and mouse single‐cell mRNA sequencing (scRNA seq) CNS datasets. Gene ontology analysis for GO:0005506 (iron ion binding) and GO:0020037 (heme binding) indicated OL cells had the highest expression levels of iron (Figure 1a) and heme (Figure 1b) binding genes amongst CNS cells. OLs were enriched in the iron storage and carrier genes *Ftl1*, *Fth1* and *Trf* (Figure 1a). Interestingly, OLs were singular in their expression of the heme binding genes fatty acid hydrolase 2 (*Fa2h*) and the heme transporter *Hrg1* (*Slc48a1*) (Figure 1b); while also expressing *Hmox2* encoding a constitutively active enzyme that liberates iron from heme (Pek et al., 2019). Using a custom list of iron/ heme metabolic genes previously assembled (Pek et al., 2019), no other iron/ heme associated genes were identified (Figure S1a).

We focused on *Hrg1* expression as it encodes a heme transporter. The Human Protein Atlas (www.proteinatlas.org) contains a meta‐analysis of 31 different datasets using the 10X Genomics platform, without pre‐enrichment of cell types and containing more than 4000 cells and 20 million reads in each study. In this unbiased dataset, we surprisingly observed that OLs expressed the highest levels of *Hrg1* (*Slc48a1*) across all human cell types including hematopoietic lineages (Figure 1c). *Hrg1* is also enriched in myelinating OLs from the Brain‐RNA‐Seq (www.brainrnaseq.org) (Zhang et al., 2014, 2016) (Figure S1b). Interestingly, astrocytes express high levels of *Hrg1* in humans compared to mice suggesting evolutionary divergence (Figure 1c,d and S1b). Other known heme transporters such as *Flvcr1* (*Mfsd7b*), *Flvcr2* (*Mfsd7c*), and *Slco2b1* showed minimal expression in oligodendroglial lineage cells (Figure S1b) and low levels across neurons and oligodendroglia (Figure S1b). Mouse (Zeisel et al., 2018) and human (Karlsson et al., 2021) scRNA seq datasets indicated *Hrg1* expression was highest in *Mbp* ^+^ and *Plp1* ^+^ mature myelinating OLs in adult CNS (Figure 1d). We analyzed single‐cell mRNA datasets from post‐natal day (P)2 and P11 mouse brain and spinal cord SPLiT‐SEQ (Split Pool Ligation‐based transcriptome sequencing) (Rosenberg et al., 2018) during OPC differentiation into mature myelinating OLs (Figure 1e). Established markers for OPCs (*Pdgfra* and *Cspg4*) and mature OLs (*Mbp*, *Plp1*, *Ermn*, and *Mog*) (Zeisel et al., 2018) as well as intermediate differentiation (*Enpp6* [Xiao et al., 2016] and *Bcas1* [Fard et al., 2017; Chavali et al., 2020]) in pre‐OLs provided references for pseudo‐timelines (Figure 1e). Against these, expression of iron transporters transferrin receptor (*Tfrc*) and *Dmt1* (*Slc11a2*) mapped to early and late differentiating OPCs, respectively (Figure 1e), whereas iron sequestering (*Fth1*, *Ftl1* and *Trf*) and heme metabolic genes (*Hrg1* and *Hmox2*) all showed maximal expression in mature myelinating OLs (Figure 1e). These findings suggested that in addition to iron import via transferrin receptor, mature OLs might also utilize the Hrg1 heme transporter to meet its iron quota.

**FIGURE 2:**
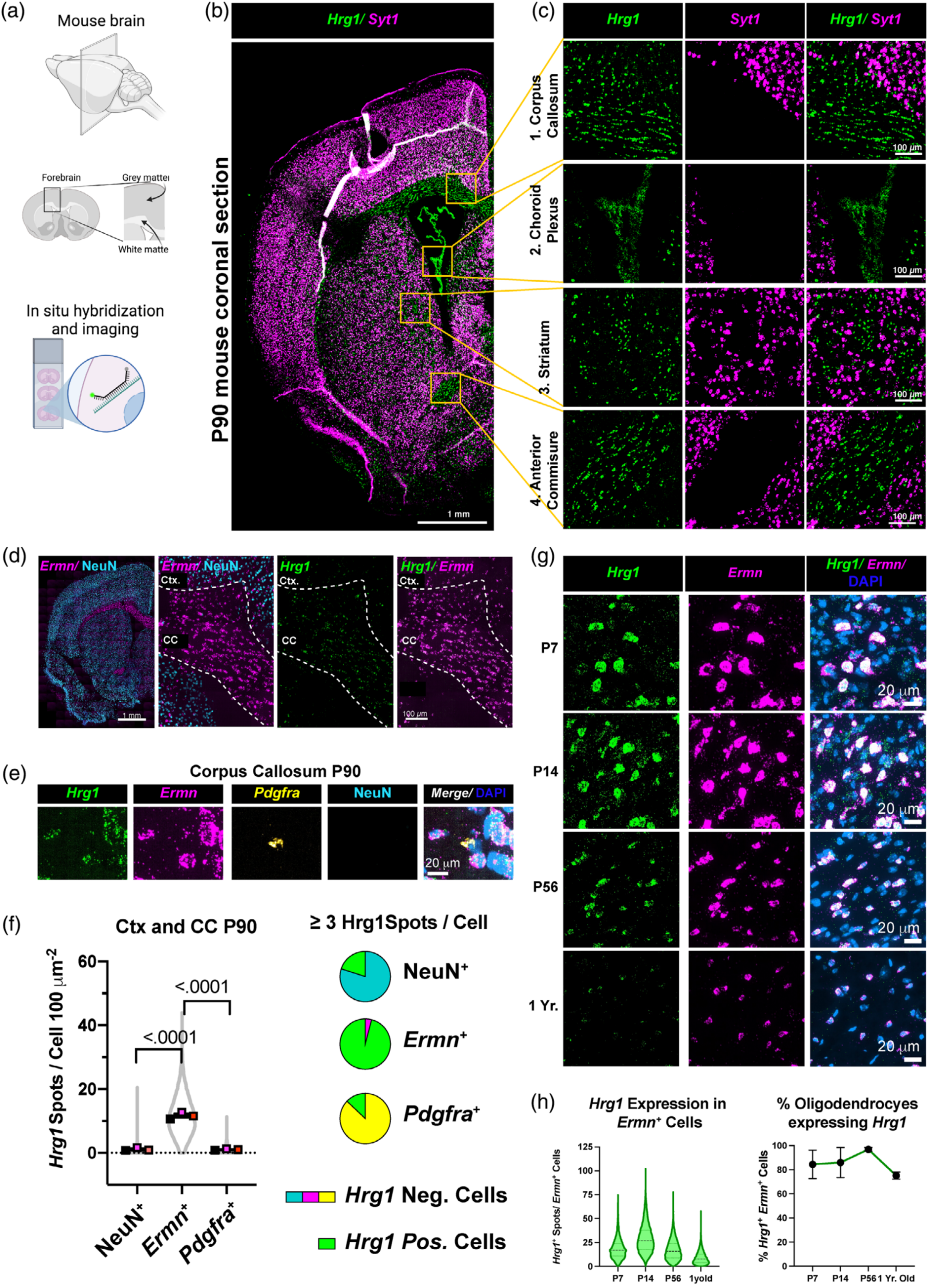
*Hrg1* is expressed in mature oligodendrocytes in vivo. (a) Schematic depicting the region from the adult (P90) mouse central nervous system (CNS) that was selected for single‐molecule fluorescence in situ hybridization (smFISH). (b) Representative stitched confocal image of an adult (P90) mouse coronal brain section, stained for neuronal *Syt1* and *Hrg1* transcripts, with four regions being selected for neuronal *Syt1* and *Hrg1* transcripts. Scale bar of 1 millimeter is shown. (c) Magnified views of selected regions from (b) showcasing white matter tracts, that is, corpus callosum (1.) and anterior commissure (4.), as well as cerebrospinal fluid‐generating cells of the choroid plexus (2.), and striatum (3.). Scale bars of 100 μ are shown. (d) Representative stitched confocal image of an adult (P90) mouse coronal brain section displaying NeuN neuronal protein and *Ermn* mature oligodendrocyte marker mRNA for gray (cerebral cortex, Ctx.) and white matter corpus callosum (CC) regions, respectively. Scale bars of 1 millimeter,100 μ are shown. (e) High magnification image of the CC illustrating *Hrg1*, *Ermn*, *Pdgfra* mRNA, NeuN protein, and DNA stained with DAPI. Scale bar of 20 μ is shown. (f) Violin plot and pie charts of *Hrg1* mRNA expression detected by smFISH in CC and cortical regions (Ctx.) of adult mouse (P90) in neurons (NeuN^+^), oligodendrocytes (*Ermn* ^+^), and oligodendrocyte precursor cells (OPCs) (*Pdgra* ^+^). (g) Representative high magnification images of *Hrg1* mRNA expression in oligodendrocytes (*Ermn* ^+^) during mouse CNS development at post‐natal day 7 (P7), P14, P56, and 1 year old in the pons. Pons was selected as oligodendrocytes are present in the same field of view at all developmental time points. No *Ermn* positive oligodendrocytes were detected in Ctx. and CC at P7. Refer to Figure S1c. Scale bars of 20 μ are shown. (h) Expression analysis of *Hrg1* mRNA in oligodendrocytes (*Ermn* ^+^) by smFISH across pons, cerebellum white matter, CC, and Ctx. at indicated time points. Left panel represent *Hrg1* mRNA spot quantification in (*Ermn* ^+^) cells with ≥7 *Ermn* spots and right panel represents the percentage of *Ermn* ^+^ cells with ≥7 *Hrg1* mRNA spots. Violin plots are represented as all data points and scatter plots the average of three biological replicates ± standard error of the mean. All unpaired *t*‐tests performed with Welch's correction, values deemed significant as *p* < .05(*), <.005(**), and <.0005(***), and ns as non‐significant. Pie charts depict cells with a threshold of ≥3 *Hrg1* spots per cell. Fluorescent images are pseudo‐colored for aid of the reader.

Using single molecule fluorescent in situ hybridization (smFISH) in adult mice with the neuronal gene *Synaptotagmin1* (*Syt1*) as a reference marker for gray matter (Figure 2b,c) we confirmed *Hrg1* transcripts in white matter tracts such as the corpus callosum (CC), striatum (Str.), and anterior commissure (AC) as well as the choroid plexus (CP), a blood cerebrospinal fluid barrier which produces cerebrospinal fluid. We quantified the co‐localization of *Hrg1* with OPCs (*Pdgfra*), mature OLs (*Ermn*), and the neuron marker (NeuN) (Figure 2d,e). While nearly all mature *Ermn* ^+^ OLs expressed *Hrg1,* its presence in NeuN^+^ neurons or *Pdgfra* ^+^ OPCs was minimal (Figure 2e,f).

Limited single‐cell RNA sequencing datasets span various developmental stages and regions, prompting us to analyze *Hrg1* mRNA expression using smFISH across cerebral cortex (Ctx.), CC, pons, and cerebellum (Cb.) (Figure 2g,h and Figure S1c). Myelination is present at post‐natal day 7 (P7) in key autonomic and locomotor regions of the pons and cerebellum, while broader CNS myelination increases at P14 and continues into adulthood (Bonetto et al., 2021). As such, we detected *Ermn* ^+^ cells at P7 only in the pons and cerebellum (Figure S1c). *Hrg1* was highly expressed at P14 in OLs, peaking at P14, a critical time for myelination and CNS iron uptake (Taylor and Morgan, 1990), and declined with age across all brain regions examined (Figure 2g,h and Figure S1c). The percentage of *Ermn* ^+^ OLs expressing Hrg1 remained high, with 96.96% cells at P56 and 75.20% at 1 year of age (Figure 2h). This persistence in OLs prompted further analysis.

### 3.2 Hrg1 proteins are localized within the myelin sheath

**FIGURE 3:**
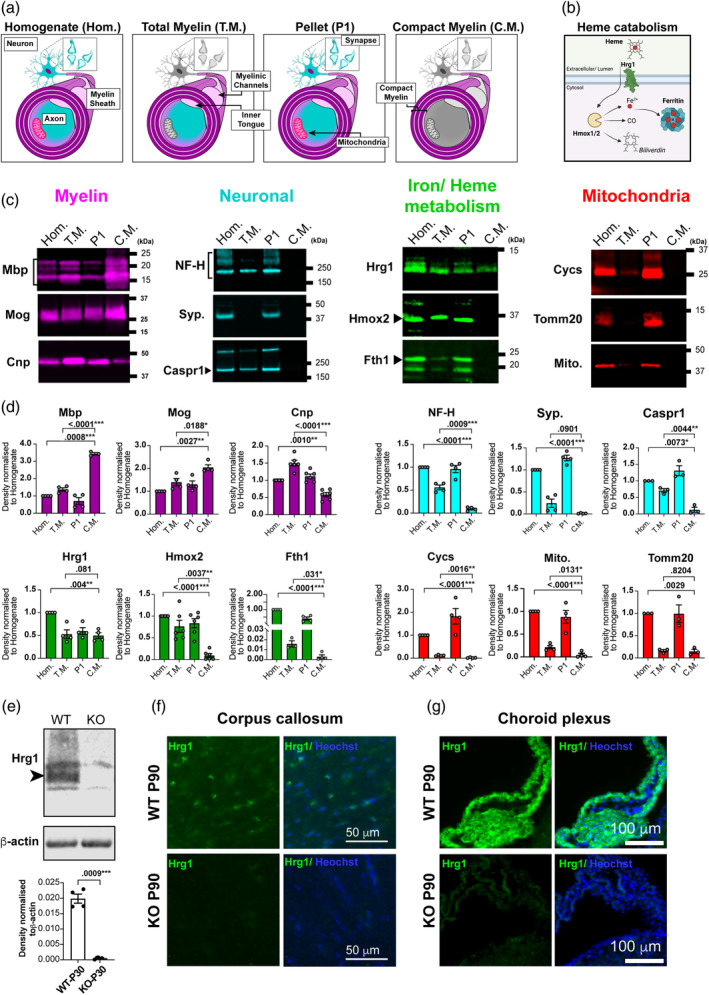
Hrg1 proteins are localized in central nervous system (CNS) myelin. (a) Schematic illustrating CNS compartments generated during the fractionation of brain homogenate (Hom.) into total myelin (T.M.), compact myelin (C.M.), and the first pellet (P1). (b) Proposed schematic of heme catabolism and iron storage relevant to this study. (c) Representative western blots of CNS fractions during myelin enrichment for myelin proteins, neuronal proteins, heme catabolism proteins as shown in (B), and mitochondrial proteins. Western blot fluorescent images are pseudo‐colored for aid of the reader. (d) Densitometry analysis of westerns in (c), normalized to their respective levels in Hom. fractions. Note the enrichment of Mbp in (C.M.), Caspr and Cnp in (T.M.), and depleted levels of neuronal proteins neurofilament‐heavy (NF‐H) and synaptophysin (Syp.), and mitochondrial proteins (Cycs, Tomm20, and mitochondrial antigen) in myelin fractions. Hrg1 is detected in all fractions, but Hmox2 and Fth1 are absent from (C.M.) Biological replicates are presented as single points on each histogram. All blots were repeated at least two times. (e) Validation of Hrg1 antibody for western blotting in mouse brain lysates from P30 wild type (WT) and Hrg1 mutants (KO), analyzed by densitometry with normalization to β‐actin levels. Western blot fluorescent images are depicted in gray scale and biological replicates are presented as single points on each histogram. All blots were repeated at least two times. (f and g) Validation of Hrg1 antibody for immunofluorescence staining in mouse brain sections from P90 WT and Hrg1 mutants in the CC (f) and choroid plexus (g). Scale bars of 50 and 100 μ are shown. Histograms error bars are ± standard error of the mean. and all unpaired *t*‐tests performed with Welch's correction, values deemed significant as *p* < .05(*), <.005(**), and <.0005(***), and ns as non‐significant. See also Figure S2a–c.

We next asked if Hrg1 proteins are present in myelin purified from brain lysates by sucrose density gradient centrifugation with osmotic stress (Erwig et al., 2019; Jahn et al., 2020). Adult rat brains, due to their larger size compared to mice, underwent homogenization followed by gradient centrifugation. This process yielded several distinct fractions: the initial brain homogenate (Hom.) collected before centrifugation, the total myelin (T.M.) layer found as the first buoyant layer at the interface of the sucrose fractions, the dense myelin‐reduced and mitochondria‐enriched fraction (P1) as the first pellet, and finally, the compact myelin fraction (C.M.) obtained after two rounds of osmotic stress and additional sucrose gradient centrifugation (see Figure 3a, Figure S2a). Fractions were examined for total protein by silver staining showing enrichment of low molecular weight proteins (Figure S2b). The majority of cholesterol in the CNS is found in myelin (Saher et al., 2005) which is also highest in myelin fractions in our study (Figure S2c).

We examined myelin (Mbp, Cnp, and Mog), neuronal (NF‐H‐200, Caspr and synaptophysin) and mitochondrial (Mitochondria antibody, Cycs and Tomm‐20) proteins by western blotting. As shown (Figure 3c,d), compact myelin proteins Mbp and Mog were highest in the C.M. fraction. The enzyme 2′,3′‐Cyclic‐nucleotide 3′‐phosphodiesterase (Cnp), a non‐compact myelin protein localized in myelinic cytoplasm channels (Snaidero et al., 2017), was enriched in the T.M. compared to C.M. fractions along with neuronal proteins of NF‐H‐200 and Caspr (Figure 3c,d); the synaptic protein synaptophysin (Syp) was absent from C.M. and T.M. fractions as expected. Previously we detected *Syp* mRNA in myelin preparations (Schirmer et al., 2019), which is consistent with *Syp* mRNA anterograde transport (Alvarez et al., 2000). Neurofilament was absent in cortical gray matter myelin sheaths (Figure S2d), distinguishing C.M. as rich in compact myelin proteins, while T.M. includes compact myelin, myelinic channel Cnp, and associated axonal components such as Caspr, NF‐H‐200, and mitochondria (Figure 3c,d), likely originating from axons.

Compact myelin accommodates small proteins, while myelinic channels house organelles and larger proteins for axonal support and local myelin maintenance (Stadelmann et al., 2019). We validated the specificity of the Hrg1 antibodies by comparative western blotting on whole CNS of *Hrg1* knock out mice (Pek et al., 2019) at P30. As shown (Figure 3e–g), Hrg1 immunoreactivity was specific for control littermates versus *Hrg1* ^*−*^/^*−*^ animals and immunofluorescence staining showed no immunoreactivity for Hrg1 in the CC or CP of mutants. Hrg1 is part of the heme catabolism machinery (Figure 3b) (Rajagopal et al., 2008; Zhang et al., 2018; Pek et al., 2019; Simmons et al., 2020; Yang et al., 2023; White et al. 2013). We detected Hrg1 in myelin fractions of T.M. and C.M., but the enzyme heme oxygenase 2 (Hmox2) which is responsible for the degradation of heme, and the iron storage protein Fth1, were present in T.M. but not C.M. fractions (Figure 3c,d), implying their presence in myelinic channels or axonal compartments that associate with the myelin sheath. These results show that heme transport, heme catabolism and iron storage proteins are all found in myelin.

### 3.3 Hrg1 proteins co‐locate with Mbp within myelin sheath

**FIGURE 4:**
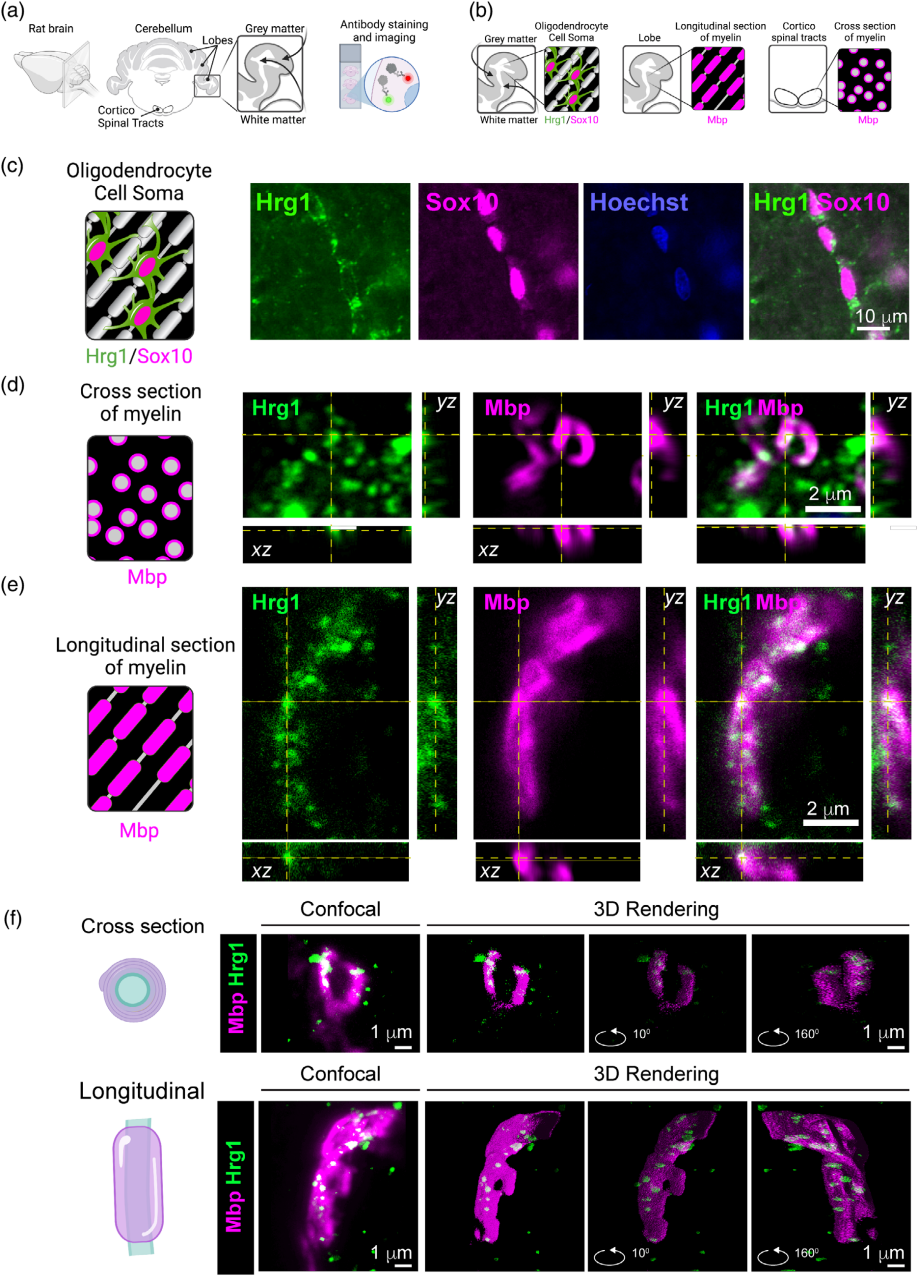
Hrg1 proteins co‐locate with Mbp within myelin sheath. (a and b) Graphical representations of mouse brain cerebellum immunofluorescence. Two main regions are analyzed for longitudinal myelin (Lobes) and cross sections of myelin in the cortico spinal tract. (c) Representative epifluorescent images of Hrg1 and Sox10 immunofluorescence in cerebellar Lobe white matter (see Figure S2f,g). Scale bar of 10 μ is shown. (d) Representative orthogonal confocal image slice of myelin sheaths at high magnification from the cortico spinal tract (cross‐sectional). Scale bar of 2 μ is shown. (e) Representative orthogonal confocal image slice of myelin sheaths at high magnification from the cerebellar lobe (longitudinal). Scale bar of 2 μ is shown. (f) Maximum intensity projection confocal image (left panels) used for 3D reconstruction using the Volume J 1.8 plugin in Fiji of myelin cross‐section (top panel) and longitudinal (bottom panel) (see Movies S1 and S2). Scale bar of 1 μ is shown.

To examine the distribution of Hrg1 within myelin, we used rat cerebellum owing to its distinct demarcation of both gray and white matter regions, as well as the presence of myelin tracts running both longitudinally in cerebellar lobes and transversely in corticospinal tracts (Figure 4a,b). Immunofluorescence analysis revealed that Hrg1 protein distribution in the cell soma of cells co‐staining with the transcription factor Sox10, a pan oligodendroglial marker, within white matter tracts (Sock and Wegner, 2021) (Figure 4c, S2f). Additionally, a subset of the Purkinje neurons also expressed Hrg1 (Figure S2g inset, arrowheads).

Comparative examination of Hrg1 alongside the compact myelin membrane marker Mbp confirmed their co‐localization (Figure S2g), substantiating the presence of Hrg1 in the myelin. We proceeded to explore the subcellular distribution of Hrg1 and Mbp proteins using high‐resolution confocal microscopy and 3D image rendering in the cerebellar white matter lobes for longitudinal myelin sheaths and cortico spinal tracts for cross sections of myelin. As depicted (Figure 4d–f, Movies S1 and S2), Hrg1 was observed to co‐localize with Mbp within the myelin sheath. The pattern of distribution of Mbp at this resolution shows an interesting helical arrangement (Figure S2d) with a calculated screw angle of 137° (Figure S2e), as determined through STED microscopy. It is important to note that Hrg1 is known to be a part of the endocytosis machinery of plasma membranes, endosomes, and lysosomes (Rajagopal et al., 2008; O'Callaghan et al., 2010), and its punctate pattern of distribution in myelin is consistent with membrane and vesicular localization.

### 3.4 Zinc mesoporphyrin uptake indicates OLs possesses a functional heme transporter

**FIGURE 5:**
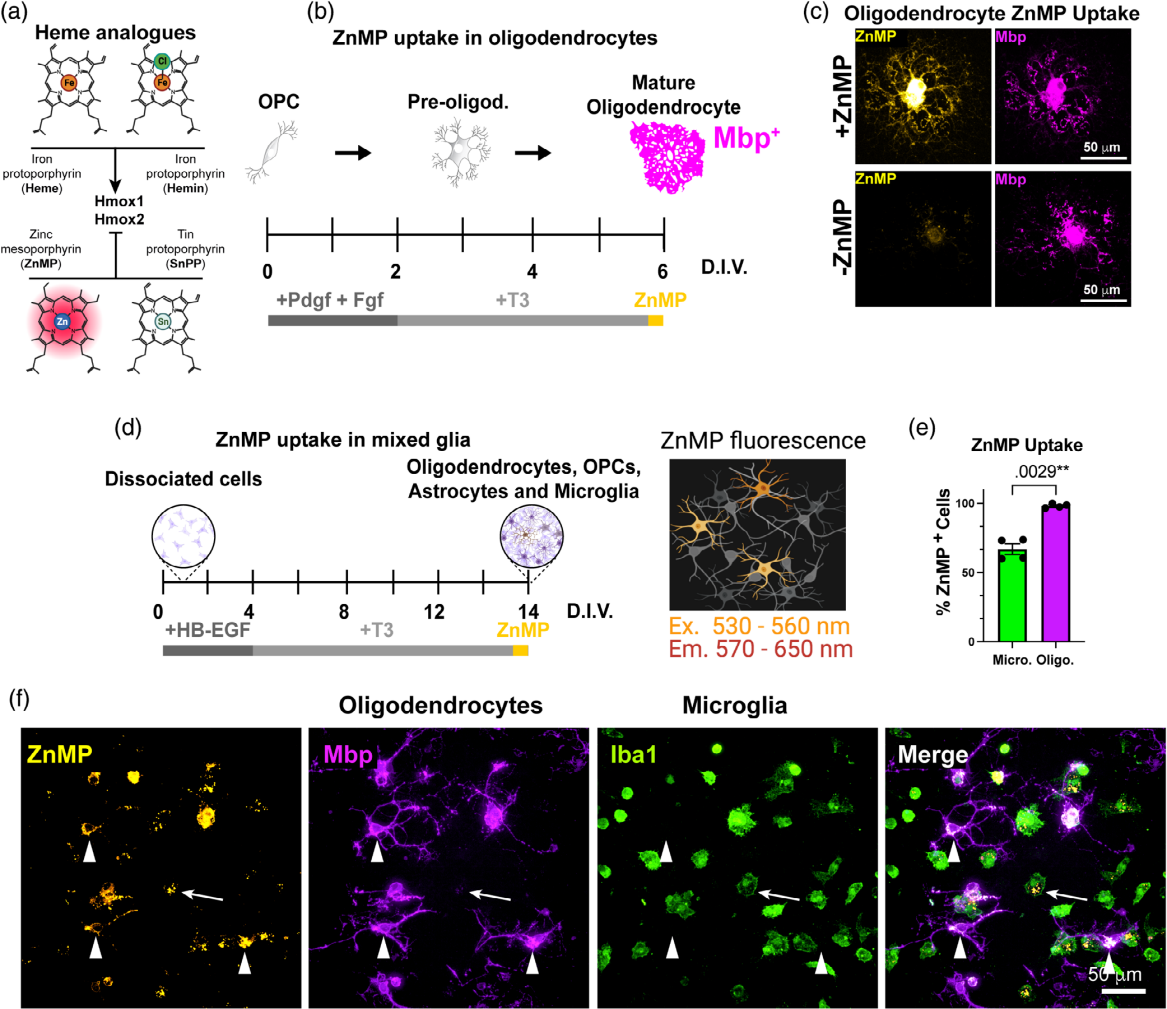
Hrg1 is a functional heme importer in oligodendrocytes. (a) Schematic of heme and heme analogues hemin (oxidized heme), zinc mesoporphyrin (ZnMP), and tin protoporphyrin (SnPP). Heme and hemin are stimulants while ZnMP and SnPP are inhibitors of heme oxygenases (Hmox1/2). (b) Schematic of method for oligodendrocyte precursor cells (OPC) differentiation, treatment with ZnMP and detection of ZnMP autofluorescence. (c) Representative maximum projection of confocal image of single Mbp (magenta) positive oligodendrocyte from rat primary cultures with or without ZnMP treatment (hot orange). Scales bars of 50 μ are shown. Note the distribution of ZnMP throughout the cytoplasm and processes and its absence from untreated cells. (d) Schematic of method for mixed glial differentiation, treatment with ZnMP, and detection of ZnMP autofluorescence. (e) Unbiased quantification of cells with ZnMP fluorescence in primary mixed glial cultures from (f). Note the higher percentage of Mbp positive oligodendrocytes (magenta histogram) containing ZnMP versus professional phagocytic microglia (green histogram). (f) Representative maximum projection of confocal image of mouse primary mixed glial cultures treated with ZnMP. Note the distribution of ZnMP (hot orange) in Mbp positive oligodendrocytes (magenta/arrowheads) and Iba1 positive microglia (green/arrows). Scales bars of 50 μ are shown. See also Figure S3a.

To address the functional aspect of Hrg1 and heme directly in OLs we used in vitro assays. Pharmacological activation and inhibition of heme metabolism can be achieved with heme analogues (Figure 5a). OPCs were isolated from rats CNS which has been historically easier than from mice (McCarthy and de Vellis, 1980; Stockley et al., 2017), and recent advancements using magnetic activated cell sorting (MACS) allows improved acute isolation using the A2B5 epitope found on OPCs from juvenile brains (Neumann et al., 2019). Differentiation of OPCs to OLs can be achieved by mitogen removal and exposure to T3 (Stockley et al., 2017; Neumann et al., 2019). We used purified rat OPCs and drove them to differentiate by growth factor withdrawal and thyroid hormone (T3) supplementation (Figure 5b) and treated with the fluorescent heme analogue zinc mesoporphyrin (ZnMP) (Figure 5c) which has been validated in worms, zebrafish, and human cells (Rajagopal et al., 2008; O'Callaghan et al., 2010; Yuan et al., 2012; Pek et al., 2019; Wang et al., 2022; Yang et al., 2023). As shown (Figure 5c), ZnMP autofluorescence is found at highest levels in the cell soma but also throughout the cell processes of Mbp^+^ OLs and the autofluorescence is absent with the omission of ZnMP treatment. To address if ZnMP uptake is specific to OLs, we used mouse mixed glial cultures, which contain microglia, astrocytes, OPCs, and OLs (Stockley et al., 2017; Jia et al., 2018); of note, such cultures are derived from post‐natal mice cortices and grown in serum free conditions with no heme supplementation; after 4 days T3 is added to drive OPC differentiation into OLs, which are allowed to mature for a further 10 days (Figure 5d). This analysis showed rapid and robust uptake of fluorescent ZnMP 15‐mins post‐treatment in 98.17% ± 0.89% of Mbp^+^ OLs (arrow heads) compared to 66.91% ± 4.38% of Iba1^+^ phagocytic microglia, and no other cells were ZnMP^+^ (Figure 5e,f, Figure S3a). These findings indicated that OLs possess a functional heme transporter.

### 3.5 Hemin (oxidized heme) rescues OPC differentiation in the setting of iron deprivation in a Hmox‐dependent manner

**FIGURE 6:**
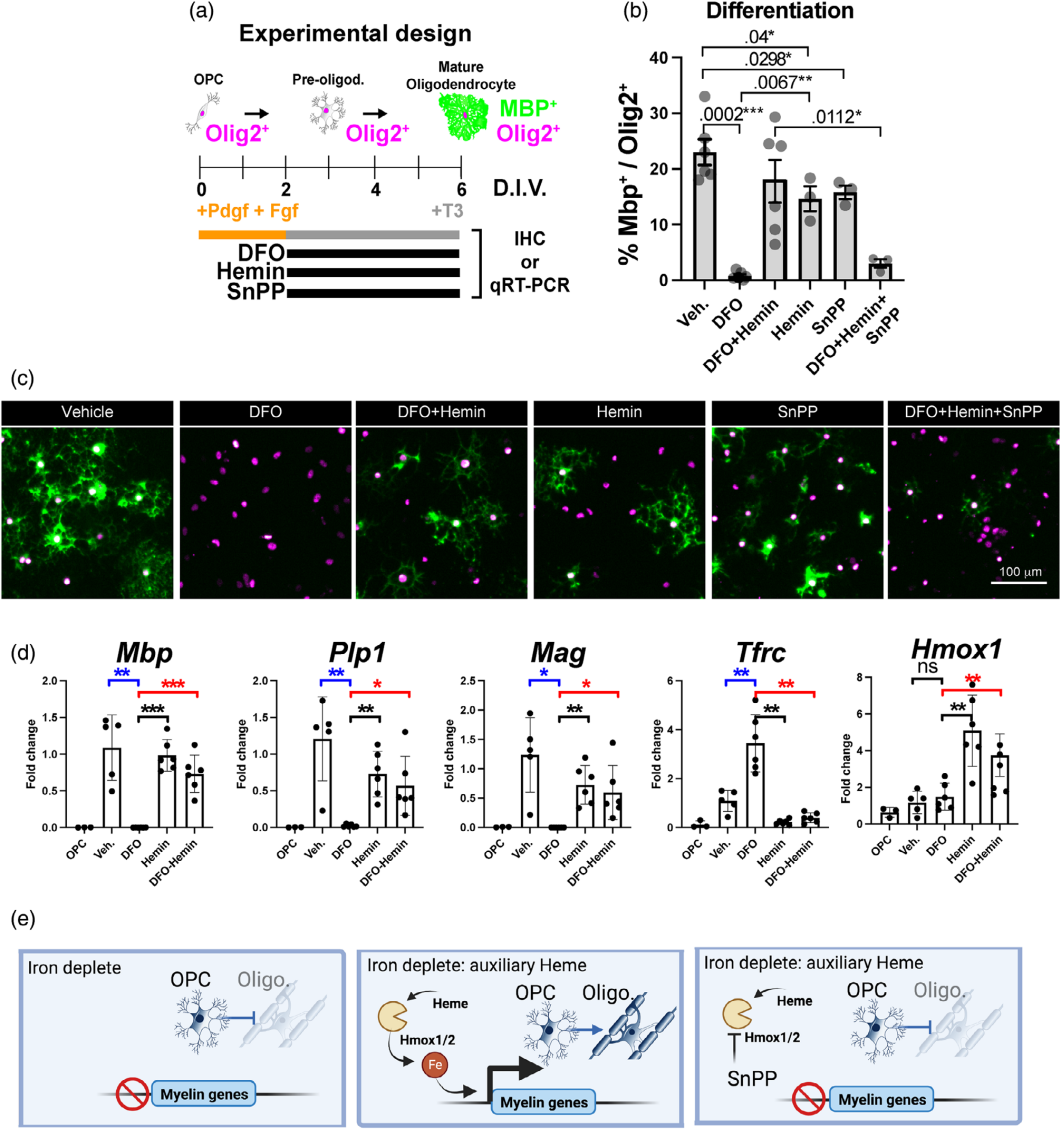
Heme is an auxiliary source of iron for oligodendrocyte precursor cells (OPC) differentiation. (a) Schematic of method for primary OPC differentiation into mature oligodendrocytes. Differentiation is initiated by growth factor (PDGF and FGF) withdrawal and supplementation with thyroid hormone T3. Black bars represent drugs used in the treatments with deferoxamine (DFO) to chelate iron, oxidized heme (hemin) as a heme surrogate and SnPP as a specific heme oxygenase inhibitor. Treated cells were analyzed by immunofluorescence or qPCR. (b) Unbiased quantification of images from (c) analyzed for the percentage of Mbp^+^ cells and normalized to the number of Olig2^+^ cells. (c) Representative epifluorescent images from cells treated with drugs stained for pan oligodendroglial marker Olig2 (magenta) and mature oligodendrocytes with Mbp (green). Quantifications depicted in (b). Scales bar1 of 100 μ are shown. Note that iron depletion with DFO blocks OPC differentiation, which is rescued by hemin treatment but requires the activity of heme oxygenases blocked by SnPP. (d) quantitative real‐time PCR (qRT‐PCR) analysis of OPCs and oligodendrocytes treated with vehicle (Veh.), DFO, Hemin, and DFO with hemin. Genes significantly responsive to iron depletion are shown as blue *p*‐values and genes responsive to hemin during iron depletion are shown as red *p*‐values. Note that iron depletion halts oligodendrocyte gene transcription (*Mbp*, *Plp1*, and *Mag*), which is rescued by hemin treatment, and conversely iron depletion elevates *Tfrc* while hemin attenuates *Tfrc* expression. *Hmox1* is also increased by hemin treatment. Biological replicates are presented as single points on each histogram and error bars are ± standard error of the mean All unpaired *t*‐tests performed with Welch's correction, values deemed significant as *p* < .05(*), <.005(**), and <.0005(***), and ns as non‐significant and fluorescent images are pseudo‐colored for aid of the reader. See also Figure S4.

**FIGURE 7:**
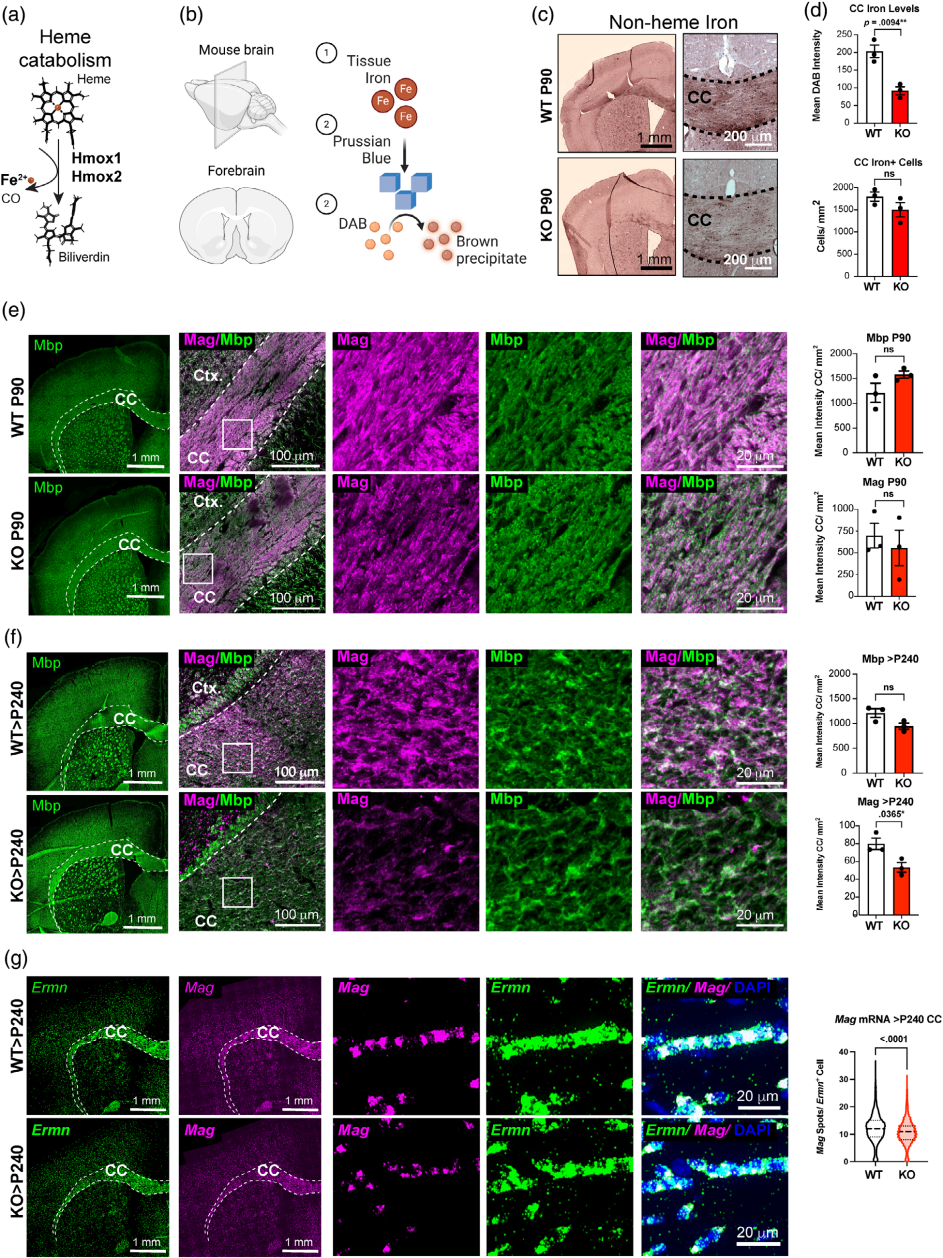
Aging *Hrg1* mutant mice show progressive reduction in Mag expression. (a) Structural schematic of heme degradation to iron, carbon monoxide (CO), and biliverdin by heme oxygenases. (b) Schematic of section Turnbull iron staining. Tissue iron in sections (1) is (2) oxidized to Prussian blue crystals by potassium ferricyanide and acts as a redox catalyst to polymerize diaminobenzidine (DAB) in the presence of H_2_0_2_. (c) Enhanced section Turnbull's staining for non‐heme iron in the central nervous system of Hrg1 deficient mice (KO) and wild type (WT) littermates at P90 (3 months old). Note the localization of brown DAB reaction indicative of iron in white matter tracts. Scales bars of 1 mm and 200 μ are shown. (d) Quantification of iron staining intensity in Hrg1 KO compared to controls is lower. At higher magnifications, this was quantified for the corpus callosum (CC) as mean DAB intensities and the number of cells with DAB staining. (e) Representative stitched confocal images from mouse coronal sections stained for Mag (Magenta) and Mbp (Green) in Hrg1 deficient mice (KO) and WT littermates at P90 (3 months old) and quantified for Mag levels in CC with scale bars of 1 mm and 100 μ. Higher magnifications of insets are shown to the right. Scale bar of 20 μ is shown. (f) Representative stitched confocal images from mouse coronal sections stained for Mag (Magenta) and Mbp (Green) in Hrg1 deficient mice (KO) and WT littermates at >P240 (>8 months old) and quantified for Mag levels in the CC with scale bars of 1 mm and 100 μ. Higher magnifications of insets are shown to the right. Scale bar of 20 μ is shown. (g) Representative stitched confocal images from mouse coronal sections stained for *Mag* (Magenta) and *Ermn* (Green) mRNA by smFISH in Hrg1 deficient mice (KO) and WT littermates at >P240 (>8 months old) and quantified for *Mag* spots per *Ermn* ^+^ cells in the CC with scale bars of 1 mm and 20 μ. Violin plots are represented as all data points and scatter plots the average of three biological replicates ± standard error of the mean. All unpaired *t*‐tests performed with Welch's correction, values deemed significant as *p* < .05(*), <.005(**), and <.0005(***), and ns as non‐significant. Pie charts depict cells with a threshold of ≥3 *Hrg1* spots per cell. Fluorescent images are pseudo‐colored for aid of the reader.

Hemin is the oxidized chlorinated form of heme (Figure 5a), and just as for heme, iron can only be liberated upon its degradation by heme oxygenase enzymes (Hmox1, 1Hmox2; Figures 3b and 7a). Heme is a well‐known driver of oxidative stress (Thorburne and Juurlink, 1996; Pek et al., 2019; Baldacchino et al., 2022; Dutt et al., 2022) and OPCs are one of the most vulnerable cells to reactive oxygen species in vitro (Thorburne and Juurlink, 1996; Stockley et al., 2017), a finding we confirmed (Figure S4b,c). We calculated EC_50_ of hemin toxicity over a 24‐h period at 5% O_2_ to be 37 and 123 μM for OPCs and OLs, respectively (Figure S3b,c), and decided to use concentrations of hemin at 1 μM. To determine a role for heme we removed iron with the chelator deferoxamine (DFO) (Hershko et al., 2001; Nobuta et al., 2019) and treated cells with the heme analogue tin‐protoporphyrin (SnPP) a potent transition state inhibitor of Hmox1/2 preventing heme catabolism (Wong et al., 2011) (Figure 6a). As shown (Figure 6b,c), DFO treatment blocked differentiation of OPCs into mature Mbp^+^ OLs, consistent with effects of iron deprivation in vivo (Lozoff et al., 2006; Isasi et al., 2022). However, addition of low dose hemin rescued the attenuated differentiation as judged by Mbp immunolabeling and upregulation of myelin transcripts *Mbp, Mag*, *and Plp1* (Figure 6b–d) in DFO treated cultures. Furthermore, while SnPP and hemin have modest effects on the differentiation of OPCs into Mbp^+^ OLs in iron‐containing conditions, we found that SnPP blocked the ability of hemin to rescue the effects of DFO treatment. We also observed transferrin receptor (*Tfrc*) expression is stimulated by iron depletion and attenuated by hemin supplementation (Figure 6d). In addition, increased expression of *Hmox1* by qPCR (Figure 6d) and the responsiveness of *Tfrc* and *Hmox1* together support successful iron depletion and heme supplementation in our experiments. Collectively, these findings indicate heme transport in oligodendroglial cells can functionally rescue iron deprivation and that catabolic breakdown of heme by Hmox1/2 is metabolically useful to OPCs during differentiation (Figure 6e).

### 3.6 Reduced iron levels in situ in *Hrg1* mutant mice

We investigated if Hrg1 is involved in iron homeostasis in OLs in vivo. The breakdown of heme via heme oxygenase (Hmox1/2 system) is shown in Figure 7a, releasing biliverdin, carbon monoxide (CO) and iron as by products. Iron can be detected using section Turnbull stain technique, as described by (Meguro et al., 2007) through the formation and precipitation of Prussian blue crystals from localized iron, which catalyze the polymerization and precipitation of diaminobenzidine (DAB) (Figure 7b). *Hrg1* deficient mice compared to controls showed reduced staining of non‐heme iron throughout white matter tracts while the number of iron‐positive cells in the CC were similar (Figure 7c,d). Inductively coupled plasma mass spectrometry (ICP‐MS) analysis of iron of whole brains from *Hrg1* ^−^/^−^ mice are significantly lower than controls (Pek et al., 2019). ICP‐MS detects total iron as both heme and non‐heme iron, while Turnbull stain technique is specific to non‐heme iron (Meguro et al., 2007). These findings indicated an essential role for Hrg1 in maintaining normal levels of iron in white matter and suggested Hrg1 could provide an alternative pathway for iron import in OLs through heme and its catabolism releasing iron.

### 3.7 Aging *Hrg1* mutant mice show progressive reduction in mag expression

Iron is an essential nutrient for myelin formation, so we next assessed myelin levels in young adult mice of post‐natal day (P90) when developmental myelination is complete and reduced iron in myelin is present. Morphological evaluations of the brain, CC, and cortical thickness (Figure S4a–c), indicated no gross differences in *Hrg1* mutant mice compared to their littermates at P90. We measured myelin levels and distribution by immunofluorescent (IF) staining for Mbp and found no difference at P90 in wild types compared to knockouts (Figure 7e). Myelin associated glycoprotein (Mag) is a minor, but significant component of myelin required for myelin attachment to neuronal axons (Li et al., 1994; Djannatian et al., 2019). A preferential loss of Mag occurs in hypoxic demyelinating lesions in multiple sclerosis (MS) CNS before loss of other myelin proteins such as MBP, PLP, and MOG (Aboul‐Enein et al., 2003), and compact myelin proteins such as Mbp and Plp are preserved in *Mag* knock out mice (Li et al., 1994). We therefore investigated levels of Mag at P90 by IF and found a minor decrease that did not achieve significance (Figure 7e). Western blots of whole brains also showed a trend in to a decrease in myelin proteins but did not attain significance (Figure S5a). However, in animals older than 8 months of age (>P240) we found a significant reduction in the levels of Mag in *Hrg1* mutants in the CC (Figure 7f); in contrast, Plp1, Mbp, and Mog levels were not significantly different (Figure 7f and Figure S4d,e).

At P90, we quantified the numbers of mature OLs using Aspa immunofluorescence in the CC, hippocampus, and cortical regions (Figure S4f–h) and found higher numbers of OLs. We confirmed these findings in the cerebral cortex using smFISH for *Emrn* ^+^ OLs (Figure S4i), while numbers of *Pdgfra* ^+^ OPCs were unchanged (Figure S4i). *Hrg1* mRNA levels were present but significantly lower in mutants (Figure S4i). We detected a statistically significant reduction in NeuN^+^ neurons (Figure S4i) implying a possible level of neurodegeneration. While these findings indicated a grossly normal phenotype, the later observation of reduced levels of Mag, which is essential for myelin adhesion (Trapp, 1990; Li et al., 1994; Voineskos et al., 2008; Djannatian et al., 2019), prompted ultrastructural analysis in *Hrg1*‐mull mice.

### 3.8 *Hrg1* function is required to maintain myelin ultrastructural integrity

**FIGURE 8:**
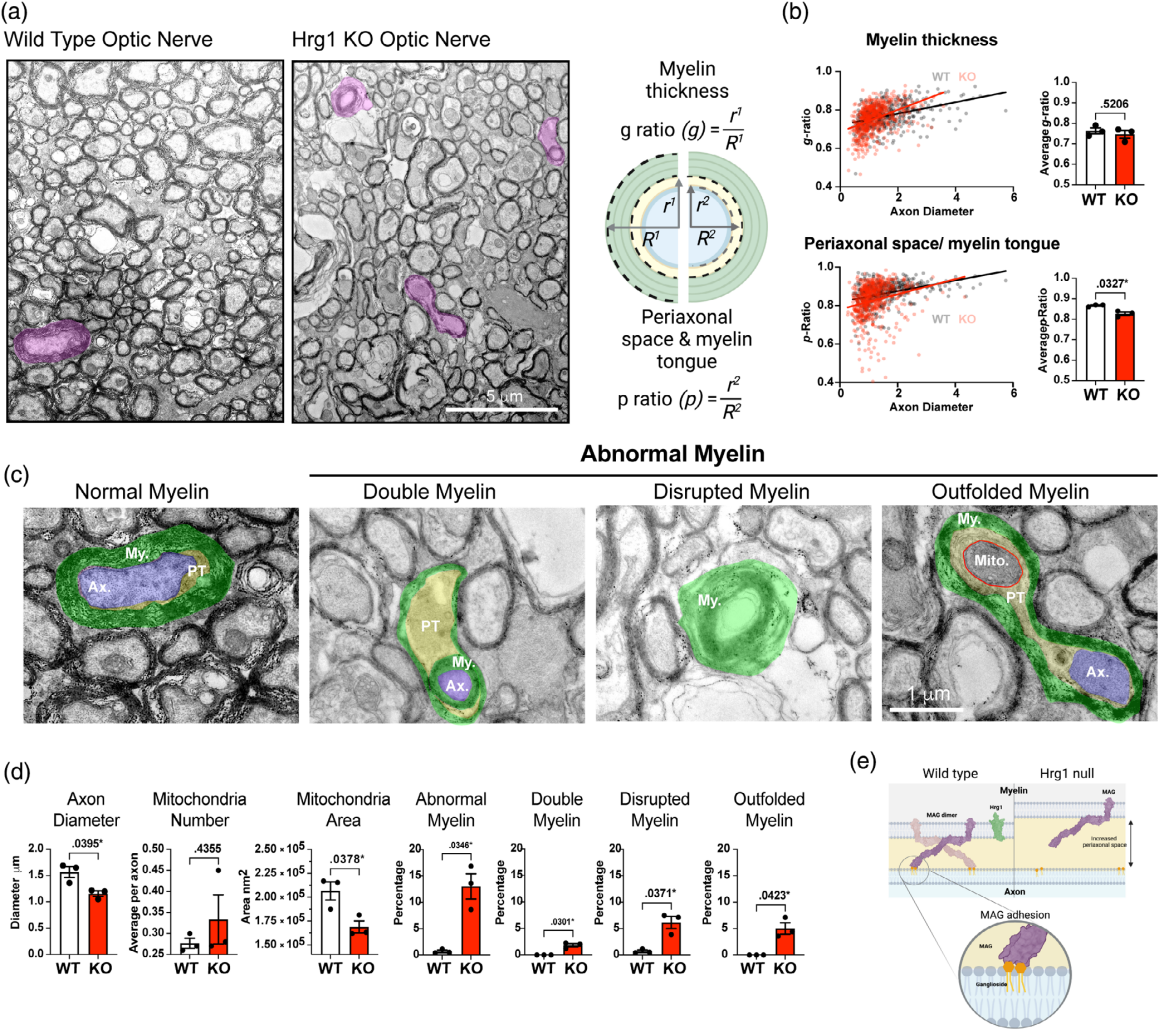
*Hrg1* function is required to maintain myelin ultrastructural integrity. (a) Electron photomicrographs of optic nerves from Hrg1 deficient mice (KO) and wild type (WT) littermates at P90, highlighting myelin types in pink. Scale bar of 5 μ is shown. (b) Schematic representation of *g*‐ratio and *p*‐ratio measurements, accompanied by scatter plots and averaged values. (c) Higher magnification of myelin types with pseudo‐coloring for myelin (green), axon (blue), periaxonal space and tongue (PT) (yellow), and mitochondria (Mito.) (circled in red). Scale bar of 1 μ is shown. (d) Morphometric analysis from ultrastructural examination reveals an increase in abnormal myelin, including double myelin, disrupted myelin, and outfolded myelin. Data presented as mean ± standard error of the mean. of three biological replicates. (e) Schematic outlining the proposed hypothesis for the loss of axonal adhesion due to reduced Mag levels in Hrg1 deficient myelin. Biological replicates are presented as single points on each histogram and error bars are ± standard error of the mean. All unpaired *t*‐tests performed with Welch's correction, values deemed significant as *p* < .05(*), <.005(**), and <.0005(***), and ns as non‐significant.

The optic nerve (ON) comprises large diameter axons which are nearly all myelinated, is thus suitable to assess myelin integrity at the ultrastructural level, and has been used previously in analysis of *Mag^−^*/*^−^* mice (Li et al., 1994; Stahon et al., 2016; Djannatian et al., 2019). Conversely, the CC has myelinated and unmyelinated axons of heterogenous axonal diameter (Blakemore and Franklin, 2008). We observed near complete myelination of axons in both wild type and *Hrg1* mutants (99.71% ± 0.21% and 96.8% ± 2.66%, respectively) (Figure 8a), assessing myelin thickness by *g*‐ratio (the ratio of the radius from the center of the axon to the inner myelin layer divided by the radius from the axon center to the outer myelin layer, Figure 8b). While we did not detect any changes in myelin thickness by *g*‐ratio measurements (Figure 8c), the periaxonal space and inner tongue of myelin was significantly larger in *Hrg1* deficient mice. To quantify this, we measured the radius from the axon center to the perimeter of the axon and the from the axon center to the inner myelin sheath and termed the ratio of these as the “*p*‐ratio” for periaxonal space thickness. *Hrg1* deficient mice had significantly reduced *p*‐ratios indicating a difference not in the thickness of myelin but an increase in the space between the axon and the myelin sheath.

As shown in Figure 8c–e, we observed three types of abnormal myelin in *Hrg1*‐mutant optic nerves, namely, (1) “double myelin” where a myelin sheath is found wrapped around another myelinated axon, (2) “disrupted myelin” also known as concentric lamellar bodies (Uranova et al., 2011) as the myelin layers with no axon, and (3) “out folded myelin” as distended myelin sheaths wrapping around an axon. These abnormal normal myelin types were significantly increased in *Hrg1* deficient animals and were reminiscent of findings in *Mag* knock out animals (Li et al., 1994; Djannatian et al., 2019), when myelin adhesion is lost from axons. Axonal mitochondria numbers remained similar in *Hrg1* mutants compared to controls, but their areas were reduced, indicating potential mitochondrial stress or issues with mitochondrial fission or fusion in the absence of Hrg1. These observations indicate significant and heterogeneous myelin ultrastructural defects in aged *Hrg1* mutant mice as depicted in Figure 8e.

## 4 DISCUSSION

### 4.1 *Slc48a1* encodes heme transporter Hrg1 that is expressed in oligodendrocyte myelin

In this study, we report for the first time that heme transporter Hrg1, encoded by *Scl48a1*, is a functional CNS myelin protein. While many heme metabolic genes are known to be expressed in the oligodendroglial lineage, consistent with the high levels of iron needed during differentiation, a surprise was our observation that *Hrg1* levels in OLs surpassed that of all other mammalian cells including hematopoietic precursors in the human protein atlas dataset. Using data from single‐cell RNA sequencing data, in situ hybridization, immunofluorescence, and cellular fractionation we show Hrg1 is expressed in mature myelinating OLs in vivo and within the myelin sheath. Indeed, these expression data indicated Hrg1 as the sole heme transporter in mammalian OLs. High resolution confocal microscopy and STED imaging reveal an interesting helical arrangement of Mbp around axons with punctate patterns of Hrg1, raising questions regarding the organization of Hrg1 with other structural proteins in myelin. Future work will attempt a detailed study of the myelin ultrastructure with immuno‐electron microscopy by 3D serial block face scanning electron microscopy and energy dispersive x‐ray spectroscopy to reveal the protein and iron arrangement in myelin.

### 4.2 Evidence that heme transport functions as an accessory source of oligodendrocyte iron

The role of Hrg1 as a heme transporter is undisputed as corroborated by data across diverse model organisms such as worms (Rajagopal et al., 2008; Yang et al., 2023), zebrafish (Zhang et al., 2018), drosophila (Wang et al., 2022), mice (Pek et al., 2019; Simmons et al., 2020), and human cells (O'Callaghan et al., 2010; Yuan et al., 2012). Several of our findings indicate direct heme transport in OLs. First, we used the classical assay of ZnMP and found that only OLs and microglia were ZnMP positive. It should be noted that microglia are professional CNS resident phagocytes but also express a potential heme transporter *Slco2b1* (Unlu et al., 2022). Despite this only 66% of microglia were positive for ZnMP uptake but nearly all mature OLs were positive for ZnMP in mixed glial cultures. No other known heme transporters in OLs are known. Second, we show that low dose oxidized heme (hemin) can rescue OPC differentiation in the presence of DFO (iron chelation), indicating that heme can function as an accessory source of iron. This effect, moreover, was inhibited by SnPP, which blocks activity of Hmox1/2 to liberate iron from heme. Lastly, we found reduced iron levels in white matter tracts of *Hrg1* mutant mice consistent with a loss of heme transport and metabolism in myelin sheaths in vivo. The later expression of *Hrg1* in the OL lineage and during aging suggests that it can be recruited as an accessory source of iron. Indeed, while later deletion of the Tfrc receptor in OPCs impairs myelination, ablation in mature OLs after myelination is established has no effect (Cheli et al., 2023). Heme is toxic to all cells, especially OPCs, which have a low threshold for oxidative stress (Thorburne and Juurlink, 1996; Stockley et al., 2017), may explain why heme import is exclusive to OLs.

The presence of porphyrins in myelin was shown nearly 80 years ago (Klüver, 1944), suggesting heme metabolism in CNS myelin and served as the basis for developing the myelin specific histochemical stain Luxol Fast Blue (Klüver and Barrera, 1953). What is the source of heme in nonpathological states? The myelin sheath and axon are metabolically coupled, myelin lipids are a source of beta‐oxidation for axons (Asadollahi et al., 2024), myelin can export lactate (Fünfschilling et al., 2012; Lee et al., 2012) and *Fth1* mRNA to axons (Mukherjee et al., 2020) for respiration and anti‐oxidant support respectively, whilst axons release neurotransmitters to myelin nodes (Micu et al., 2017). The localization of Hrg1 in compact and non‐compact myelin, and the altered periaxonal space in *Hrg1* ^−/−^ optic nerves suggests the possibility of an axonal source. An intriguing hypothesis would be that axonal heme (e.g., derived from mitochondrial cytochrome) is removed locally by Hrg1 channels in myelin, providing iron for OLs. Together, these findings indicate that Hrg1 comprises an accessory pathway, essential for iron import via heme in the oligodendroglial lineage.

### 4.3 *Hrg1* function is required for myelin integrity as well as regulation of Mag

Our findings indicate that iron is critical for OPC differentiation and expression of myelin transcripts *Mbp, Plp1*, and *Mag* in vitro. The expression of *Hrg1* coincides with maximal myelination in the CNS at P14 in myelinating OLs, but remaining high in OLs even when myelination is complete. This is consistent with roles for Tfrc iron in OPCs import during development but not in mature adult OLs (Cheli et al., 2023), implying a potential iron switch during OPC differentiation from non‐heme iron (transferrin bound iron) to heme iron. We detected an age associated decline in Mag protein by immunofluorescent staining and *Mag* mRNA by smFISH, but not for other myelin proteins. Mag is a critical component of myelin, required for myelin adhesion to neuronal axons (Bartsch et al., 1989; Trapp, 1990). Mag, a glycosylated protein of 100 kDa mass, is larger than conventional myelin proteins (typically <35 kDa) that spans the myelin sheath and the axon within the periaxonal space (Trapp, 1990; Li et al., 1994; Djannatian et al., 2019). At the ultrastructural level, loss of Mag causes enlargement of the periaxonal space due to loss of Mag binding to axonal gangliosides, and the formation of outfolded myelin and double myelin (Li et al., 1994; Djannatian et al., 2019). These unusual myelin structures are thought to be a result of myelin sheaths from adjacent myelin nodes growing over each other during development (Djannatian et al., 2019). Interestingly, we detected identical ultrastructural alterations in adult *Hrg1* mutants; namely, double myelin, outfolded myelin, and enlarged periaxonal spaces, as observed in *Mag* knockouts. Also, we found reduced levels of Mag in older *Hrg1* deficient mice versus other myelin proteins Mbp, Plp1, and Mog. Prior studies have shown loss of Mag in acute demyelinated lesions in MS before loss of other myelin proteins such as Plp and Mog (Aboul‐Enein et al., 2003). In these lesions, hypoxia is believed to be a driver of CNS damage, which can lead to dysfunctional axonal mitochondria. Loss of Hrg1 does not cause anemia when placed on normal iron diets as their hematocrit levels are unchanged (Pek et al., 2019; Simmons et al., 2020). Given the decline in OL *Hrg1* expression from P14 to 1 year old animals it is interesting that myelin disturbances in *Hrg1* mutant mice do not manifest earlier. We did observe increased OLs and decreased neuronal numbers in the cerebral cortex of P90 *Hrg1* deficient mice. Heme is toxic to all cells (Chambers et al., 2021), and hemin is toxic to OPCs as well as OLs (Figure S3b,c). We hypothesize that a breakdown in heme transport from axon to myelin through loss of Hrg1 causes neuronal loss and concomitant oligodendrocyte survival. We lack sufficient evidence to conclusively prove this and suggest fate mapping by EdU treatment of Hrg1 deficient mice at different developmental time points combined with the use of conditional deletion of Hrg1 to address this.

Axonal mitochondria, the primary energy source for active neurons, are vulnerable to oxidative damage (Bergaglio et al., 2021). Mitochondrial dysfunction may disrupt axo‐glial communication and metabolic coupling, especially as myelin support axonal energy through lipid and lactate (Fünfschilling et al., 2012; Lee et al., 2012; Asadollahi et al., 2024). Although we have not identified the link between Hrg1 function and Mag levels, our findings support a model in which chronic late deprivation of iron availability in *Hrg1* ^*−*^/^*−*^ mice alter Mag levels in mature myelin, thereby altering adhesion and interaction with axonal ligands. Future work should address *Hrg1* function in experimental models of demyelination, especially in context of iron deficiency, and the impact on remyelination and *Mag* regulation.

### 4.4 Might Hrg1 have pathological roles in neurological conditions?

Myelin dysfunction is a driver of pathological hallmarks of Alzheimer's disease (Depp et al., 2023). Dysregulated iron levels are associated with many neurodegenerative disorders (Rouault, 2013; Ward et al., 2014; Möller et al., 2019), and iron has a pleiotropic role in OLs, where it is essential for myelin development, but also toxic at high doses (Healy et al., 2018; Nobuta et al., 2019; Baldacchino et al., 2022). OLs have the highest levels of iron in the CNS (Connor et al., 1990; Meguro et al., 2007; Todorich et al., 2009; Badaracco et al., 2010) and prior studies show demyelinated lesions in MS are depleted in iron, except at the lesion rim where iron storage genes are elevated (Schirmer et al., 2019), a finding associated with disease progression (Absinta et al., 2019). In the rare genetic disorder, Pelizaeus‐Merzbacher disease (PMD) (Nobuta et al., 2019; Elitt et al., 2020), we observed that *PLP1* mutations lead to a state of ferroptosis in OLs, which could be attenuated by iron chelation (Nobuta et al., 2019). Administration of iron chelators to Hrg1 null mice is of interest, however, low iron diets exacerbate morbidity in Hrg1 knockout (KO) models due to severe impairment in erythrophagocytosis and iron recycling (Pek et al., 2019). It is anticipated that iron chelators may pose similar challenges in Hrg1 null mice. This type of experiment draws attention to the need to develop conditional deletion of Hrg1 in OLs to elucidate the role of iron and heme metabolism in myelin, which is beyond the scope of this study.

Intracerebral hemorrhage often damages the myelin rich basal ganglia (Jiang et al., 2019), and it has been shown that chronic infusion of heme oxygenase inhibitors attenuates myelin damage (Gu et al., 2016). These considerations support future work to determine whether Hrg1 might predispose the OL to heme accumulation and iron toxicity in genetic and injury conditions of haemorrhagic stroke or serum leakage associated with neuro‐inflammatory lesions.

## AUTHOR CONTRIBUTIONS

John. H. Stockley and David H. Rowitch conceived the original idea and John. H. Stockley, Adrien M. Vaquie, and David H. Rowitch wrote the manuscript. John. H. Stockley and Adrien M. Vaquie performed all tissue immunofluorescence, oligodendrocyte cell cultures with assistance from Staffan Holmqvist and Daniel Yamamoto All qPCR work was by Adrien M. Vaquie smFISH experiments were performed by Theresa Bartels and Gregory D. Jordan and analyzed by Adrien M. Vaquie, Gregory D. Jordan, and John. H. Stockley. Iron tissue staining was performed by John. H. Stockley and Simon Gunter and Zhaoyang Xu designed, performed, and analyzed bioinformatic data. Myfanwy Hill euthanized and perfused rat tissues, Rini H. Pek, Ian G. Chambers, and Andrew S. Rock prepared mouse tissue. John. H. Stockley, Andrew S. Rock, and Chao Zhao prepared tissue for transmission electron microscopy and Scott Dillon and John. H. Stockley performed tem. Guy Lam performed silver staining of gels and John. H. Stockley performed all western blots and myelin fractionation data.

## FUNDING INFORMATION

This work was supported by funding from the European Research Council Grant (ID: 789054) (to David H. Rowitch), Wellcome Trust (ID: 108139/Z/15/Z) (to David H. Rowitch) and Dr. Miriam and Sheldon G. Adelson Medical Research Foundation (to David H. Rowitch, Robin J.M. Franklin) and R01DK125740 to Iqbal Hamza. This work was supported by the NIHR Cambridge Biomedical Research Centre (NIHR203312). Views expressed are those of the authors and not necessarily those of the NIHR or the Department of Health and Social Care.

## CONFLICT OF INTEREST STATEMENT

Iqbal Hamza is the President and Founder of Rakta Therapeutics Inc. (Maryland), a company involved in the development of heme transporter‐related diagnostics. He declares no other competing financial interests. The remaining authors declare no competing interests.

## Supporting information


**Figure S1.** Iron metabolic gene expression in CNS cells.
Related to Figures 1 and 2.
(a) Heat map displaying the expression of custom list of genes (rows) associated with iron metabolism from Pek et al. (2019) across six CNS cell types (columns). Expression levels were normalized and shown in log scale.
(b) Data from Brain‐RNA‐Seq (www.brainrnaseq.org) (Zhang et al., 2014; 2016) showing expression of main heme transporters across human and mouse CNS cell types.
(c) Quantification of Hrg1 expression by smFISH in *Ermn* ^+^ oligodendrocytes across different ages of mice in the cerebellum (Cb.), corpus callosum (CC), pons and cerebral cortex (Ctx.). Note, no mature *Ermn* ^+^ oligodendrocytes are detectable in the CC or Ctx. at P7.

**Figure S2.** Biochemical and histochemical analysis of CNS myelin.
Related to Figures 3 and 4.
(a) Typical myelin fractionations generated from sucrose gradient fractionation of rat CNS. The first round of centrifugation reveals a buoyant layer (left centrifuge tube) that is off white yellow in color termed as total myelin (T.M.). After further centrifugation and washing of the total myelin, the buoyant layer is white with a fluffy consistency, which when pelleted and washed is compact myelin (C.M.).
(b) 15 ◻g of myelin fractions were separated on Tris‐Tricine gels and stained for total protein by silver method. Note the enrichment of low molecular weight proteins in T.M. and C.M. lanes.
(c) Amplex red total cholesterol assay performed on purified lipids from myelin fractions. Cholesterol was measured against a standard curve. Four biological replicates are presented as single points on each histogram. All unpaired *t*‐tests performed with Welch's correction, values deemed significant as *p* < .05(*), <.005(**), and <.0005(***), and ns as non‐significant.
(d) STED imaging of CNS myelin stained for Mbp (magenta) and neurofilament heavy (NF) (cyan). Left pane is maximum projection and individual *z*‐stacks through the projection are from left to right. Orthogonal view of a single *z* plane is shown on the right. Scale bars of 1 μ are shown. Note the helical distribution of Mbp around the axon, which is illustrated.
(e) Measurement of angles of Mbp turning relative to axon represented as a violin plot from a minimum of four biological replicates. Angles measured using Fiji and analyzed in GraphPad Prism and data shown as a violin plot with mean and error bars represent S.E.M.
(f) Low magnification epifluorescent images for Hrg1 (green), Sox10 (magenta), and nuclei (blue) in cerebellar white matter regions. Scale bars of 500 and 50 μ are shown.
(g) Epifluorescent images of Hrg1 (green) and Mbp (magenta) immhunohistochemistry localization in cerebellar white matter regions. Hrg1 is also present in the cell soma of large cells adjacent to white matter tract consistent with Purkinje neurons. Scale bars of 500 and 100 μ are shown. All fluorescent images are pseudo‐colored for aid of the reader.

**Figure S3.** Metalloporphyrin treatment of cell in vitro.
Related to Figure 5.
(a) Image of oligodendrocytes (Mbp), microglia (Iba1) ZnMP (hot orange) uptake compared to all other cells present detected with Hoechst. Supporting data to Figure 5d–f. Scale bar of 50 μ is shown.
(b) Live/Dead assay of primary rat oligodendrocyte precursor cells (OPCs) top panel and primary rat oligodendrocytes (bottom panel) cultured at 21% atmospheric oxygen and treated with 20 ◻M hemin or 20 ◻M iron as found in ferric citrate for 24 h. Live cells contain Calcein AM dye and dead cells are ethidium homodimer (Eth. H.) positive. Cells were imaged and quantified using a semi‐automated pipeline in Harmony software. Data are presented as histograms showing mean ± S.E.M. from an 5 to 6 biological replicates and scale bars of 100 μ are shown.
(c) MTT viability assay of OPCs (green) and oligodendrocytes (red) cultured at 5% oxygen and treated with increasing concentrations of hemin (oxidized heme). EC_50_ values were calculated using GraphPad prism v 9 from four biological replicates. Note the increased in EC_50_ values for oligodendrocytes compared to OPCs.

**Figure S4.** Morphological and cellular assessment in Hrg1 deficient animals.
Related to Figure 7.
(a) Fixed mouse brains from Hrg1 deficient and littermate controls from P90 animals.
(b) Histograms of manual analysis of corpus callosum (CC) morphology for gross area and maximum thickness in Hrg1 deficient and littermate controls from P90 animals.
(c) Histograms of manual analysis of cortical thickness in the mouse barrel cortex in Hrg1 deficient and littermate controls from P90 animals.
(d) Representative stitched confocal images from mouse coronal sections stained for Plp1 (Yellow) in Hrg1 deficient mice (KO) and wild type (WT) littermates at >P240 (8 months of age or older) and quantified for Plp1 levels in the CC. Data presented as mean ± S.E.M. of 3 biological replicates. Scale bar of 1 mm is shown. Note the levels of Plp1 are not significantly different between Hrg1 deficient mice and controls.
(e) Representative stitched confocal images from mouse coronal sections stained for Mog (Gray scale) in Hrg1 deficient mice (KO) and wild type (WT) littermates at >P240 (8 months of age or older) and quantified for Mog levels in the CC. Data presented as mean ± S.E.M. of 3 biological replicates. Scale bar of 1 mm is shown. Note the levels of Mog are not significantly different between Hrg1 deficient mice and controls.
(f) Maximum projection of confocal image from the mouse CC in Hrg1 deficient mice (KO) and wild type (WT) littermates at P90 (3 months old) for the mature oligodendroglial marker Aspartoacylase (Aspa) shown as magenta. Cells were quantified manually and normalized to the area per mm^2^ and data presented as mean ± S.E.M. of 3 biological replicates. Scale bar of 100 μ is shown.
(g and h) Immunohistochemistry for Apsa (magenta), Mbp (green) and nuclei (blue) in the hippocampus (g) and retrosplenial cortex (h) from Hrg1 deficient and littermate controls at age P90. Manual quantification of Aspa cell numbers are shown in their respective histograms. Scale bars of 200 μ are shown.
(i) Fluorescent in situ hybridization and immunohistochemistry for mature oligodendrocytes (*Ermn*), oligodendrocyte precursor cells (*Pdgfra*) and *Hrg1* transcripts neurons (NeuN) and nuclei (Hoechst). Cell numbers were quantified using an automated pipeline in Harmony and are shown below. Scale bars of 100 μ are shown.
Biological replicates are presented as single points on each histogram. All unpaired *t*‐tests performed with Welch's correction, values deemed significant as *p* < .05(*), <.005(**), and <.0005(***), and ns as non‐significant. Fluorescent images are pseudo‐colored for aid of the reader.

**Figure S5.** Myelin protein levels in Adult *Hrg1* mutant.
Related to Figure 7.
(a) Representative western blots of whole CNS homogenates from P30 (1 month old) Hrg1 deficient mice (KO) and wild type (WT) littermates for myelin proteins Mbp, Cnp, and Mag, neurofilament‐heavy (NF‐H), heme catabolism enzymes (Hmox2 and Hmox1), and β‐actin as a loading control. Densitometric analyses of westerns normalized to respective β‐actin levels are shown below, representing means ± S.E.Ms. Western blot fluorescent images are depicted in gray scale and biological replicates of 4–5 are presented as single points on each histogram. All blots were repeated at least two times. Arrowheads and square bracket indicate the bands used for western blot quantifications.

**Table S1.** Supporting Information.

**Movies S1 and S2.** 3D Rendering of Hrg1 and Mbp co‐localization in myelin.
Related to Figure 4.
(S1) Longitudinal 3D rendering of confocal image of myelin sheath with Mbp (magenta) and Hrg1 (green) rotated about axis. Scale bar of 1 μ is shown.
(S2) Cross‐section 3D rendering of confocal image of myelin sheath with Mbp (magenta) and Hrg1 (green) rotated about axis. Scale bar of 1 μ is shown.

## Data Availability

The data that support the findings of this study are available from the corresponding author upon reasonable request.

## References

[glia24641-bib-0001] Aboul‐Enein, F. , Rauschka, H. , Kornek, B. , Stadelmann, C. , Stefferl, A. , Brück, W. , Lucchinetti, C. , Schmidbauer, M. , Jellinger, K. , & Lassmann, H. (2003). Preferential loss of myelin‐associated glycoprotein reflects hypoxia‐like white matter damage in stroke and inflammatory brain diseases. Journal of Neuropathology and Experimental Neurology, 62, 25–33. 12528815 10.1093/jnen/62.1.25

[glia24641-bib-0002] Absinta, M. , Sati, P. , Masuzzo, F. , Nair, G. , Sethi, V. , Kolb, H. , Ohayon, J. , Wu, T. , Cortese, I. C. M. , & Reich, D. S. (2019). Association of Chronic Active Multiple Sclerosis Lesions with Disability in Vivo. JAMA Neurology, 76, 1474–1483. 31403674 10.1001/jamaneurol.2019.2399 PMC6692692

[glia24641-bib-0003] Alvarez, J. , Giuditta, A. , & Koenig, E. (2000). Protein synthesis in axons and terminals: Significance for maintenance, plasticity and regulation of phenotype. With a critique of slow transport theory. Progress in Neurobiology, 62, 1–62. 10821981 10.1016/s0301-0082(99)00062-3

[glia24641-bib-0004] Andreini, C. , Putignano, V. , Rosato, A. , & Banci, L. (2018). The human iron‐proteome. Metallomics, 10, 1223–1231. 30095136 10.1039/c8mt00146d

[glia24641-bib-0005] Asadollahi, E. , Trevisiol, A. , Saab, A. S. , Looser, Z. J. , Dibaj, P. , Ebrahimi, R. , Kusch, K. , Ruhwedel, T. , Möbius, W. , Jahn, O. , Lee, J. Y. , Don, A. S. , Khalil, M.‐A. , Hiller, K. , Baes, M. , Weber, B. , Abel, E. D. , Balabio, A. , Popko, B. , … Nave, K.‐A. (2024). Oligodendroglial fatty acid metabolism as a central nervous system energy reserve. Nature Neuroscience, 27, 1934–1944. 39251890 10.1038/s41593-024-01749-6 PMC11452346

[glia24641-bib-0006] Badaracco, M. E. , Siri, M. V. R. , & Pasquini, J. M. (2010). Oligodendrogenesis: The role of iron. BioFactors, 36, 98–102. 20336710 10.1002/biof.90

[glia24641-bib-0007] Baldacchino, K. , Peveler, W. J. , Lemgruber, L. , Smith, R. S. , Scharler, C. , Hayden, L. , Komarek, L. , Lindsay, S. L. , Barnett, S. C. , Edgar, J. M. , Linington, C. , & Thümmler, K. (2022). Myelinated axons are the primary target of hemin‐mediated oxidative damage in a model of the central nervous system. Experimental Neurology, 354, 114113. 35569511 10.1016/j.expneurol.2022.114113

[glia24641-bib-0008] Bartsch, U. , Kirchhoff, F. , & Schachner, M. (1989). Immunohistological localization of the adhesion molecules L1, N‐CAM, and MAG in the developing and adult optic nerve of mice. The Journal of Comparative Neurology, 284, 451–462. 2474006 10.1002/cne.902840310

[glia24641-bib-0009] Bayraktar, O. A. , Bartels, T. , Holmqvist, S. , Kleshchevnikov, V. , Martirosyan, A. , Polioudakis, D. , Ben Haim, L. , Young, A. M. , Batiuk, M. Y. , & Prakash, K. (2020). Astrocyte layers in the mammalian cerebral cortex revealed by a single‐cell in situ transcriptomic map. Nature Neuroscience, 23, 500–509. 32203496 10.1038/s41593-020-0602-1 PMC7116562

[glia24641-bib-0010] Bergaglio, T. , Luchicchi, A. , & Schenk, G. J. (2021). Engine failure in Axo‐myelinic signaling: A potential key player in the pathogenesis of multiple sclerosis. Frontiers in Cellular Neuroscience, 15, 610295. 33642995 10.3389/fncel.2021.610295 PMC7902503

[glia24641-bib-0011] Blakemore, W. F. , & Franklin, R. J. M. (2008). Remyelination in experimental models of toxin‐induced demyelination. Current Topics in Microbiology and Immunology, 318, 193–212. 18219819 10.1007/978-3-540-73677-6_8

[glia24641-bib-0012] Bonetto, G. , Belin, D. , & Káradóttir, R. T. (2021). Myelin: A gatekeeper of activity‐dependent circuit plasticity? Science, 374, eaba6905. 34618550 10.1126/science.aba6905

[glia24641-bib-0013] Chambers, I. G. , Willoughby, M. M. , Hamza, I. , & Reddi, A. R. (2021). One ring to bring them all and in the darkness bind them: The trafficking of heme without deliverers. Biochimica et Biophysica Acta (BBA)—Molecular Cell Research, 1868, 118881. 33022276 10.1016/j.bbamcr.2020.118881 PMC7756907

[glia24641-bib-0014] Chavali, M. , Ulloa‐Navas, M. J. , Pérez‐Borredá, P. , Garcia‐Verdugo, J. M. , McQuillen, P. S. , Huang, E. J. , & Rowitch, D. H. (2020). Wnt‐dependent Oligodendroglial‐endothelial interactions regulate White matter vascularization and attenuate injury. Neuron, 108, 1130–1145. 33086038 10.1016/j.neuron.2020.09.033 PMC7769920

[glia24641-bib-0015] Cheli, V. T. , Santiago González, D. A. , Marziali, L. N. , Zamora, N. N. , Guitart, M. E. , Spreuer, V. , Pasquini, J. M. , & Paez, P. M. (2018). The divalent metal transporter 1 (DMT1) is required for iron uptake and Normal development of oligodendrocyte progenitor cells. The Journal of Neuroscience, 38, 9142–9159. 30190412 10.1523/JNEUROSCI.1447-18.2018 PMC6199407

[glia24641-bib-0016] Cheli, V. T. , Santiago González, D. A. , Wan, R. , Rosenblum, S. L. , Denaroso, G. E. , Angeliu, C. G. , Smith, Z. , Wang, C. , & Paez, P. M. (2023). Transferrin receptor is necessary for proper oligodendrocyte iron homeostasis and development. The Journal of Neuroscience, 43, 3614–3629. 36977582 10.1523/JNEUROSCI.1383-22.2023 PMC10198458

[glia24641-bib-0017] Chrast, R. , Saher, G. , Nave, K.‐A. , & Verheijen, M. H. G. (2011). Lipid metabolism in myelinating glial cells: Lessons from human inherited disorders and mouse models. Journal of Lipid Research, 52, 419–434. 21062955 10.1194/jlr.R009761 PMC3035679

[glia24641-bib-0018] Connor, J. R. , Menzies, S. L. , St Martin, S. M. , & Mufson, E. J. (1990). Cellular distribution of transferrin, ferritin, and iron in normal and aged human brains. Journal of Neuroscience Research, 27, 595–611. 2079720 10.1002/jnr.490270421

[glia24641-bib-0019] Depp, C. , Sun, T. , Sasmita, A. O. , Spieth, L. , Berghoff, S. A. , Nazarenko, T. , Overhoff, K. , Steixner‐Kumar, A. A. , Subramanian, S. , Arinrad, S. , Ruhwedel, T. , Möbius, W. , Göbbels, S. , Saher, G. , Werner, H. B. , Damkou, A. , Zampar, S. , Wirths, O. , Thalmann, M. , … Nave, K.‐A. (2023). Myelin dysfunction drives amyloid‐β deposition in models of Alzheimer's disease. Nature, 618, 349–357. 37258678 10.1038/s41586-023-06120-6 PMC10247380

[glia24641-bib-0020] Djannatian, M. , Timmler, S. , Arends, M. , Luckner, M. , Weil, M.‐T. , Alexopoulos, I. , Snaidero, N. , Schmid, B. , Misgeld, T. , Möbius, W. , Schifferer, M. , Peles, E. , & Simons, M. (2019). Two adhesive systems cooperatively regulate axon ensheathment and myelin growth in the CNS. Nature Communications, 10, 4794–4815. 10.1038/s41467-019-12789-z PMC6805957 31641127

[glia24641-bib-0021] Dutt, S. , Hamza, I. , & Bartnikas, T. B. (2022). Molecular mechanisms of iron and heme metabolism. Annual Review of Nutrition, 42, 311–335. 10.1146/annurev-nutr-062320-112625 PMC9398995 35508203

[glia24641-bib-0022] Elitt, M. S. , Barbar, L. , Shick, H. E. , Powers, B. E. , Maeno‐Hikichi, Y. , Madhavan, M. , Allan, K. C. , Nawash, B. S. , Gevorgyan, A. S. , Hung, S. , Nevin, Z. S. , Olsen, H. E. , Hitomi, M. , Schlatzer, D. M. , Zhao, H. T. , Swayze, A. , LePage, D. F. , Jiang, W. , Conlon, R. A. , … Tesar, P. J. (2020). Suppression of proteolipid protein rescues Pelizaeus‐Merzbacher disease. Nature, 585, 397–403. 32610343 10.1038/s41586-020-2494-3 PMC7810164

[glia24641-bib-0023] Erwig, M. S. , Hesse, D. , Jung, R. B. , Uecker, M. , Kusch, K. , Tenzer, S. , Jahn, O. , & Werner, H. B. (2019). Myelin: Methods for purification and proteome analysis. Methods in Molecular Biology, 1936, 37–63. 30820892 10.1007/978-1-4939-9072-6_3

[glia24641-bib-0024] Fard, M. K. , van der Meer, F. , Sánchez, P. , Cantuti‐Castelvetri, L. , Mandad, S. , Jäkel, S. , Fornasiero, E. F. , Schmitt, S. , Ehrlich, M. , Starost, L. , Kuhlmann, T. , Sergiou, C. , Schultz, V. , Wrzos, C. , Brück, W. , Urlaub, H. , Dimou, L. , Stadelmann, C. , & Simons, M. (2017). BCAS1 expression defines a population of early myelinating oligodendrocytes in multiple sclerosis lesions. Science Translational Medicine, 9, eaam7816. 10.1126/scitranslmed.aam7816 PMC7116798 29212715

[glia24641-bib-0025] Folch, J. , Lees, M. , & Sloane Stanley, G. H. (1957). A simple method for the isolation and purification of total lipides from animal tissues. The Journal of Biological Chemistry, 226, 497–509. 13428781

[glia24641-bib-0026] Freeman, M. R. , & Rowitch, D. H. (2013). Evolving concepts of gliogenesis: A look way back and ahead to the next 25 years. Neuron, 80, 613–623. 24183014 10.1016/j.neuron.2013.10.034 PMC5221505

[glia24641-bib-0027] Fünfschilling, U. , Supplie, L. M. , Mahad, D. , Boretius, S. , Saab, A. S. , Edgar, J. , Brinkmann, B. G. , Kassmann, C. M. , Tzvetanova, I. D. , Möbius, W. , Diaz, F. , Meijer, D. , Suter, U. , Hamprecht, B. , Sereda, M. W. , Moraes, C. T. , Frahm, J. , Goebbels, S. , & Nave, K.‐A. (2012). Glycolytic oligodendrocytes maintain myelin and long‐term axonal integrity. Nature, 485, 517–521. 22622581 10.1038/nature11007 PMC3613737

[glia24641-bib-0028] Gu, Y. , Gong, Y. , Liu, W.‐Q. , Keep, R. F. , Xi, G. , & Hua, Y. (2016). Zinc protoporphyrin attenuates White matter injury after intracerebral hemorrhage. Acta Neurochirurgica. Supplement, 121, 199–202. 26463948 10.1007/978-3-319-18497-5_35

[glia24641-bib-0029] Healy, S. , McMahon, J. , & FitzGerald, U. (2018). UPR induction prevents iron accumulation and oligodendrocyte loss in ex vivo cultured hippocampal slices. Frontiers in Neuroscience, 12, 969. 30618588 10.3389/fnins.2018.00969 PMC6305600

[glia24641-bib-0030] Hershko, C. , Konijn, A. M. , Nick, H. P. , Breuer, W. , Cabantchik, Z. I. , & Link, G. (2001). ICL670A: A new synthetic oral chelator: Evaluation in hypertransfused rats with selective radioiron probes of hepatocellular and reticuloendothelial iron stores and in iron‐loaded rat heart cells in culture. Blood, 97, 1115–1122. 11159545 10.1182/blood.v97.4.1115

[glia24641-bib-0031] Isasi, E. , Figares, M. , Abudara, V. , & Olivera‐Bravo, S. (2022). Gestational and lactational iron deficiency anemia impairs myelination and the neurovascular unit in infant rats. Molecular Neurobiology, 59, 3738–3754. 35381889 10.1007/s12035-022-02798-3

[glia24641-bib-0032] Jahn, O. , Siems, S. B. , Kusch, K. , Hesse, D. , Jung, R. B. , Liepold, T. , Uecker, M. , Sun, T. , & Werner, H. B. (2020). The CNS myelin proteome: Deep profile and persistence after post‐mortem delay. Frontiers in Cellular Neuroscience, 14, 239. 32973451 10.3389/fncel.2020.00239 PMC7466725

[glia24641-bib-0033] Jia, M. , Shi, Z. , Yan, X. , Xu, L. , Dong, L. , Li, J. , Wang, Y. , Yang, S. , & Yuan, F. (2018). Insulin and heparin‐binding epidermal growth factor‐like growth factor synergistically promote astrocyte survival and proliferation in serum‐free medium. Journal of Neuroscience Methods, 307, 240–247. 29890195 10.1016/j.jneumeth.2018.06.002

[glia24641-bib-0034] Jiang, Y.‐B. , Wei, K.‐Y. , Zhang, X.‐Y. , Feng, H. , & Hu, R. (2019). White matter repair and treatment strategy after intracerebral hemorrhage. CNS Neuroscience & Therapeutics, 25, 1113–1125. 31578825 10.1111/cns.13226 PMC6823871

[glia24641-bib-0035] Kaiser, T. , Allen, H. M. , Kwon, O. , Barak, B. , Wang, J. , He, Z. , Jiang, M. , & Feng, G. (2021). MyelTracer: A semi‐automated software for myelin g‐ratio quantification. eNeuro, 8, ENEURO.0558‐20.2021. 10.1523/ENEURO.0558-20.2021 PMC8298095 34193510

[glia24641-bib-0036] Karlsson, M. , Zhang, C. , Méar, L. , Zhong, W. , Digre, A. , Katona, B. , Sjöstedt, E. , Butler, L. , Odeberg, J. , Dusart, P. , Edfors, F. , Oksvold, P. , Feilitzen von, K. , Zwahlen, M. , Arif, M. , Altay, O. , Li, X. , Ozcan, M. , Mardinoglu, A. , … Lindskog, C. (2021). A single‐cell type transcriptomics map of human tissues. Science Advances, 7, eabh2169. 10.1126/sciadv.abh2169 PMC8318366 34321199

[glia24641-bib-0037] Karlsson, U. , & Schultz, R. L. (1965). Fixation of the central nervous system from electron microscopy by aldehyde perfusion. I. Preservation with aldehyde perfusates versus direct perfusion with osmium tetroxide with special reference to membranes and the extracellular space. Journal of Ultrastructure Research, 12, 160–186. 14289426 10.1016/s0022-5320(65)80014-4

[glia24641-bib-0038] Klüver, H. , & Barrera, E. (1953). A method for the combined staining of cells and fibers in the nervous system. Journal of Neuropathology and Experimental Neurology, 12, 400–403. 13097193 10.1097/00005072-195312040-00008

[glia24641-bib-0039] Klüver, H. (1944). On naturally occurring porphyrins in the central nervous system. Science, 99, 482–484. 17792234 10.1126/science.99.2581.482

[glia24641-bib-0040] Lee, Y. , Morrison, B. M. , Li, Y. , Lengacher, S. , Farah, M. H. , Hoffman, P. N. , Liu, Y. , Tsingalia, A. , Jin, L. , Zhang, P.‐W. , Pellerin, L. , Magistretti, P. J. , & Rothstein, J. D. (2012). Oligodendroglia metabolically support axons and contribute to neurodegeneration. Nature, 487, 443–448. 22801498 10.1038/nature11314 PMC3408792

[glia24641-bib-0041] Li, C. , Tropak, M. B. , Gerlai, R. , Clapoff, S. , Abramow‐Newerly, W. , Trapp, B. , Peterson, A. , & Roder, J. (1994). Myelination in the absence of myelin‐associated glycoprotein. Nature, 369, 747–750. 7516497 10.1038/369747a0

[glia24641-bib-0042] Lozoff, B. , Beard, J. , Connor, J. , Barbara, F. , Georgieff, M. , & Schallert, T. (2006). Long‐lasting neural and behavioral effects of iron deficiency in infancy. Nutrition Reviews, 64, 34–43. 10.1301/nr.2006.may.S34-S43 PMC1540447 16770951

[glia24641-bib-0043] McCarthy, K. D. , & de Vellis, J. (1980). Preparation of separate astroglial and oligodendroglial cell cultures from rat cerebral tissue. The Journal of Cell Biology, 85, 890–902. 6248568 10.1083/jcb.85.3.890 PMC2111442

[glia24641-bib-0044] Meguro, R. , Asano, Y. , Odagiri, S. , Li, C. , Iwatsuki, H. , & Shoumura, K. (2007). Nonheme‐iron histochemistry for light and electron microscopy: A historical, theoretical and technical review. Archives of Histology and Cytology, 70, 1–19. 17558140 10.1679/aohc.70.1

[glia24641-bib-0045] Micu, I. , Plemel, J. R. , Caprariello, A. V. , Nave, K.‐A. , & Stys, P. K. (2017). Axo‐myelinic neurotransmission: A novel mode of cell signalling in the central nervous system. Nature Reviews. Neuroscience, 19, 58. 10.1038/nrn.2017.166 29238086

[glia24641-bib-0046] Möller, H. E. , Bossoni, L. , Connor, J. R. , Crichton, R. R. , Does, M. D. , Ward, R. J. , Zecca, L. , Zucca, F. A. , & Ronen, I. (2019). Iron, myelin, and the brain: Neuroimaging meets neurobiology. Trends in Neurosciences, 42, 384–401. 31047721 10.1016/j.tins.2019.03.009

[glia24641-bib-0047] Mukherjee, C. , Kling, T. , Russo, B. , Miebach, K. , Kess, E. , Schifferer, M. , Pedro, L. D. , Weikert, U. , Fard, M. K. , Kannaiyan, N. , Rossner, M. , Aicher, M.‐L. , Goebbels, S. , Nave, K.‐A. , Krämer‐Albers, E.‐M. , Schneider, A. , & Simons, M. (2020). Oligodendrocytes provide antioxidant defense function for neurons by secreting ferritin heavy chain. Cell Metabolism, 32, 259–272. 32531201 10.1016/j.cmet.2020.05.019 PMC7116799

[glia24641-bib-0048] Neumann, B. , Baror, R. , Zhao, C. , Segel, M. , Dietmann, S. , Rawji, K. S. , Foerster, S. , McClain, C. R. , Chalut, K. , van Wijngaarden, P. , & Franklin, R. J. M. (2019). Metformin restores CNS remyelination capacity by rejuvenating aged stem cells. Cell Stem Cell, 25, 473–485.e8. 31585093 10.1016/j.stem.2019.08.015 PMC6863391

[glia24641-bib-0049] Nobuta, H. , Yang, N. , Ng, Y. H. , Marro, S. G. , Sabeur, K. , Chavali, M. , Stockley, J. H. , Killilea, D. W. , Walter, P. B. , & Zhao, C. (2019). Oligodendrocyte death in Pelizaeus‐Merzbacher disease is rescued by iron chelation. Cell Stem Cell, 25, 531–541. 31585094 10.1016/j.stem.2019.09.003 PMC8282124

[glia24641-bib-0051] O'Callaghan, K. M. , Ayllon, V. , O'Keeffe, J. , Wang, Y. , Cox, O. T. , Loughran, G. , Forgac, M. , & O'Connor, R. (2010). Heme‐binding protein HRG‐1 is induced by insulin‐like growth factor I and associates with the vacuolar H+‐ATPase to control endosomal pH and receptor trafficking. The Journal of Biological Chemistry, 285, 381–391. 19875448 10.1074/jbc.M109.063248 PMC2805445

[glia24641-bib-0052] Pek, R. H. , Yuan, X. , Rietzschel, N. , Zhang, J. , Jackson, L. , Nishibori, E. , Ribeiro, A. , Simmons, W. , Jagadeesh, J. , Sugimoto, H. , Alam, M. Z. , Garrett, L. , Haldar, M. , Ralle, M. , Phillips, J. D. , Bodine, D. M. , & Hamza, I. (2019). Hemozoin produced by mammals confers heme tolerance. eLife, 8, e49503. 31571584 10.7554/eLife.49503 PMC6773446

[glia24641-bib-0053] Rajagopal, A. , Rao, A. U. , Amigo, J. , Tian, M. , Upadhyay, S. K. , Hall, C. , Uhm, S. , Mathew, M. K. , Fleming, M. D. , Paw, B. H. , Krause, M. , & Hamza, I. (2008). Haem homeostasis is regulated by the conserved and concerted functions of HRG‐1 proteins. Nature, 453, 1127–1131. 18418376 10.1038/nature06934 PMC4058867

[glia24641-bib-0054] Rosenberg, A. B. , Roco, C. M. , Muscat, R. A. , Kuchina, A. , Sample, P. , Yao, Z. , Graybuck, L. T. , Peeler, D. J. , Mukherjee, S. , Chen, W. , Pun, S. H. , Sellers, D. L. , Tasic, B. , & Seelig, G. (2018). Single‐cell profiling of the developing mouse brain and spinal cord with split‐pool barcoding. Science, 360, 176–182. 29545511 10.1126/science.aam8999 PMC7643870

[glia24641-bib-0055] Rouault, T. A. (2013). Iron metabolism in the CNS: Implications for neurodegenerative diseases. Nature Reviews. Neuroscience, 14, 551–564. 23820773 10.1038/nrn3453

[glia24641-bib-0056] Saher, G. , Brügger, B. , Lappe‐Siefke, C. , Möbius, W. , Tozawa, R.‐I. , Wehr, M. C. , Wieland, F. , Ishibashi, S. , & Nave, K.‐A. (2005). High cholesterol level is essential for myelin membrane growth. Nature Neuroscience, 8, 468–475. 15793579 10.1038/nn1426

[glia24641-bib-0057] Schirmer, L. , Velmeshev, D. , Holmqvist, S. , Kaufmann, M. , Werneburg, S. , Jung, D. , Vistnes, S. , Stockley, J. H. , Young, A. , & Steindel, M. (2019). Neuronal vulnerability and multilineage diversity in multiple sclerosis. Nature, 573, 75–82. 31316211 10.1038/s41586-019-1404-z PMC6731122

[glia24641-bib-0058] Simmons, W. R. , Wain, L. , Toker, J. , Jagadeesh, J. , Garrett, L. J. , Pek, R. H. , Hamza, I. , & Bodine, D. M. (2020). Normal iron homeostasis requires the transporter SLC48A1 for efficient heme‐iron recycling in mammals. Frontiers in Genome Editing, 2, 8. 34713217 10.3389/fgeed.2020.00008 PMC8525403

[glia24641-bib-0059] Snaidero, N. , Velte, C. , Myllykoski, M. , Raasakka, A. , Ignatev, A. , Werner, H. B. , Erwig, M. S. , Möbius, W. , Kursula, P. , Nave, K.‐A. , & Simons, M. (2017). Antagonistic functions of MBP and CNP establish cytosolic channels in CNS myelin. Cell Reports, 18, 314–323. 28076777 10.1016/j.celrep.2016.12.053 PMC5263235

[glia24641-bib-0060] Sock, E. , & Wegner, M. (2021). Using the lineage determinants Olig2 and Sox10 to explore transcriptional regulation of oligodendrocyte development. Developmental Neurobiology, 81, 892–901. 34480425 10.1002/dneu.22849

[glia24641-bib-0061] Stadelmann, C. , Timmler, S. , Barrantes‐Freer, A. , & Simons, M. (2019). Myelin in the central nervous system: Structure, function, and pathology. Physiological Reviews, 99, 1381–1431. 31066630 10.1152/physrev.00031.2018

[glia24641-bib-0062] Stahon, K. E. , Bastian, C. , Griffith, S. , Kidd, G. J. , Brunet, S. , & Baltan, S. (2016). Age‐related changes in axonal and mitochondrial ultrastructure and function in White matter. The Journal of Neuroscience, 36, 9990–10001. 27683897 10.1523/JNEUROSCI.1316-16.2016 PMC5039264

[glia24641-bib-0063] Stockley, J. H. , Evans, K. , Matthey, M. , Volbracht, K. , Agathou, S. , Mukanowa, J. , Burrone, J. , & Káradóttir, R. T. (2017). Surpassing light‐induced cell damage in vitro with novel cell culture media. Scientific Reports, 7, 1–11. 28405003 10.1038/s41598-017-00829-x PMC5429800

[glia24641-bib-0064] Taylor, E. M. , & Morgan, E. H. (1990). Developmental changes in transferrin and iron uptake by the brain in the rat. Brain Research. Developmental Brain Research, 55, 35–42. 2208639 10.1016/0165-3806(90)90103-6

[glia24641-bib-0065] Thorburne, S. K. , & Juurlink, B. H. (1996). Low glutathione and high iron govern the susceptibility of oligodendroglial precursors to oxidative stress. Journal of Neurochemistry, 67, 1014–1022. 8752107 10.1046/j.1471-4159.1996.67031014.x

[glia24641-bib-0066] Todorich, B. , Pasquini, J. M. , Garcia, C. I. , Paez, P. M. , & Connor, J. R. (2009). Oligodendrocytes and myelination: The role of iron. Glia, 57, 467–478. 18837051 10.1002/glia.20784

[glia24641-bib-0067] Trapp, B. D. (1990). Myelin‐associated glycoprotein. Location and potential functions. Annals of the New York Academy of Sciences, 605, 29–43. 1702602 10.1111/j.1749-6632.1990.tb42378.x

[glia24641-bib-0068] Unlu, G. , Prizer, B. , Erdal, R. , Yeh, H.‐W. , Bayraktar, E. C. , & Birsoy, K. (2022). Metabolic‐scale gene activation screens identify SLCO2B1 as a heme transporter that enhances cellular iron availability. Molecular Cell, 82, 2832–2843. 35714613 10.1016/j.molcel.2022.05.024 PMC9356996

[glia24641-bib-0069] Uranova, N. A. , Vikhreva, O. V. , Rachmanova, V. I. , & Orlovskaya, D. D. (2011). Ultrastructural alterations of myelinated fibers and oligodendrocytes in the prefrontal cortex in schizophrenia: A postmortem morphometric study. Schizophrenia Research and Treatment, 2011, 325789. 22937264 10.1155/2011/325789 PMC3420756

[glia24641-bib-0070] Voineskos, A. N. , de Luca, V. , Bulgin, N. L. , van Adrichem, Q. , Shaikh, S. , Lang, D. J. , Honer, W. G. , & Kennedy, J. L. (2008). A family‐based association study of the myelin‐associated glycoprotein and 2′,3′‐cyclic nucleotide 3′‐phosphodiesterase genes with schizophrenia. Psychiatric Genetics, 18, 143–146. 18496213 10.1097/YPG.0b013e3282fa1874

[glia24641-bib-0071] Wang, Z. , Zeng, P. , & Zhou, B. (2022). Identification and characterization of a heme exporter from the MRP family in Drosophila melanogaster. BMC Biology, 20, 126–218. 35655259 10.1186/s12915-022-01332-0 PMC9161523

[glia24641-bib-0072] Ward, R. J. , Zucca, F. A. , Duyn, J. H. , Crichton, R. R. , & Zecca, L. (2014). The role of iron in brain ageing and neurodegenerative disorders. Lancet Neurology, 13, 1045–1060. 25231526 10.1016/S1474-4422(14)70117-6 PMC5672917

[glia24641-bib-0073] White, C. , Yuan, X. , Schmidt, P. J. , Bresciani, E. , Samuel, T. K. , Campagna, D. , Hall, C. , Bishop, K. , Calicchio, M. L. , Lapierre, A. , Ward, D. M. , Liu, P. , Fleming, M. D. , & Hamza, I. (2013). HRG1 is essential for heme transport from the phagolysosome of macrophages during erythrophagocytosis. Cell Metabolism, 17, 61–70. 23395172 10.1016/j.cmet.2013.01.005 PMC3582031

[glia24641-bib-0074] Wolf, F. A. , Hamey, F. K. , Plass, M. , Solana, J. , Dahlin, J. S. , Göttgens, B. , Rajewsky, N. , Simon, L. , & Theis, F. J. (2019). PAGA: Graph abstraction reconciles clustering with trajectory inference through a topology preserving map of single cells. Genome Biology, 20, 59. 30890159 10.1186/s13059-019-1663-x PMC6425583

[glia24641-bib-0075] Wong, R. J. , Vreman, H. J. , Schulz, S. , Kalish, F. S. , Pierce, N. W. , & Stevenson, D. K. (2011). In vitro inhibition of heme oxygenase isoenzymes by metalloporphyrins. Journal of Perinatology, 31(Suppl 1), S35–S41. 21448202 10.1038/jp.2010.173

[glia24641-bib-0076] Xiao, L. , Ohayon, D. , McKenzie, I. A. , Sinclair‐Wilson, A. , Wright, J. L. , Fudge, A. D. , Emery, B. , Li, H. , & Richardson, W. D. (2016). Rapid production of new oligodendrocytes is required in the earliest stages of motor‐skill learning. Nature Neuroscience, 19, 1210–1217. 27455109 10.1038/nn.4351 PMC5008443

[glia24641-bib-0077] Yang, Y. , Zhou, J. , Wu, F. , Tong, D. , Chen, X. , Jiang, S. , Duan, Y. , Yao, C. , Wang, T. , Du, A. , Gasser, R. B. , & Ma, G. (2023). Haem transporter HRG‐1 is essential in the barber's pole worm and an intervention target candidate. PLoS Pathogens, 19, e1011129. 36716341 10.1371/journal.ppat.1011129 PMC9910794

[glia24641-bib-0078] Yu, G. S. , Steinkirchner, T. M. , Rao, G. A. , & Larkin, E. C. (1986). Effect of prenatal iron deficiency on myelination in rat pups. The American Journal of Pathology, 125, 620–624. 2432794 PMC1888477

[glia24641-bib-0079] Yuan, X. , Protchenko, O. , Philpott, C. C. , & Hamza, I. (2012). Topologically conserved residues direct heme transport in HRG‐1‐related proteins. The Journal of Biological Chemistry, 287, 4914–4924. 22174408 10.1074/jbc.M111.326785 PMC3281596

[glia24641-bib-0080] Zeisel, A. , Hochgerner, H. , Lönnerberg, P. , Johnsson, A. , Memic, F. , van der Zwan, J. , Häring, M. , Braun, E. , Borm, L. E. , La Manno, G. , Codeluppi, S. , Furlan, A. , Lee, K. , Skene, N. , Harris, K. D. , Hjerling‐Leffler, J. , Arenas, E. , Ernfors, P. , Marklund, U. , & Linnarsson, S. (2018). Molecular architecture of the mouse nervous system. Cell, 174, 999–1014. 30096314 10.1016/j.cell.2018.06.021 PMC6086934

[glia24641-bib-0081] Zhang, J. , Chambers, I. , Yun, S. , Phillips, J. , Krause, M. , & Hamza, I. (2018). Hrg1 promotes heme‐iron recycling during hemolysis in the zebrafish kidney. PLoS Genetics, 14, e1007665. 30248094 10.1371/journal.pgen.1007665 PMC6171960

[glia24641-bib-0082] Zhang, Y. , Chen, K. , Sloan, S. A. , Bennett, M. L. , Scholze, A. R. , O'Keeffe, S. , Phatnani, H. P. , Guarnieri, P. , Caneda, C. , Ruderisch, N. , Deng, S. , Liddelow, S. A. , Zhang, C. , Daneman, R. , Maniatis, T. , Barres, B. A. , & Wu, J. Q. (2014). An RNA‐sequencing transcriptome and splicing database of glia, neurons, and vascular cells of the cerebral cortex. The Journal of Neuroscience, 34, 11929–11947. 25186741 10.1523/JNEUROSCI.1860-14.2014 PMC4152602

[glia24641-bib-0083] Zhang, Y. , Sloan, S. A. , Clarke, L. E. , Caneda, C. , Plaza, C. A. , Blumenthal, P. D. , Vogel, H. , Steinberg, G. K. , Edwards, M. S. B. , Li, G. , Duncan, J. A. , Cheshier, S. H. , Shuer, L. M. , Chang, E. F. , Grant, G. A. , Gephart, M. G. H. , & Barres, B. A. (2016). Purification and characterization of progenitor and mature human astrocytes reveals transcriptional and functional differences with mouse. Neuron, 89, 37–53. 26687838 10.1016/j.neuron.2015.11.013 PMC4707064

