# Supplementary figures and images for: Oligodendrocyte Slc48a1 (Hrg1) encodes a functional heme transporter required for myelin integrity
Source: Glia. 2024 Nov 6;73(2):399–421. doi: 10.1002/glia.24641 (PMC11662986; doi:10.1002/glia.24641)

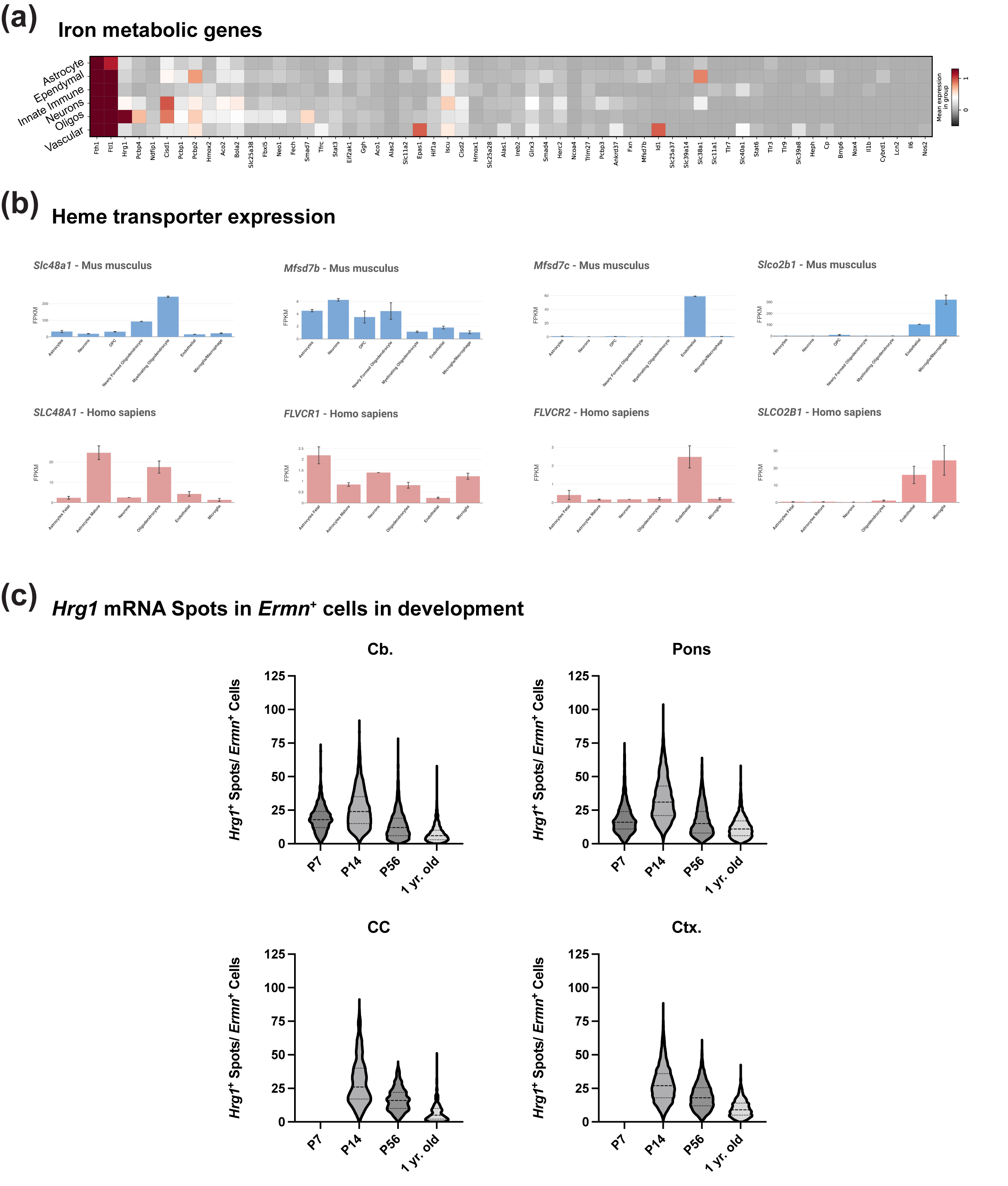

Supplement: Supplementary file 1 — Figure S1. Iron metabolic gene expression in CNS cells. Related to Figures 1 and 2. (a) Heat map displaying the expression of custom list of genes (rows) associated with iron metabolism from Pek et al. (2019) across six CNS cell types (columns). Expression levels were normalized and shown in log scale. (b) Data from Brain‐RNA‐Seq (www.brainrnaseq.org) (Zhang et al., 2014; 2016) showing expression of main heme transporters across human and mouse CNS cell types. (c) Quantification of Hrg1 expression by smFISH in Ermn + oligodendrocytes across different ages of mice in the cerebellum (Cb.), corpus callosum (CC), pons and cerebral cortex (Ctx.). Note, no mature Ermn + oligodendrocytes are detectable in the CC or Ctx. at P7. [file GLIA-73-399-s007.tif]

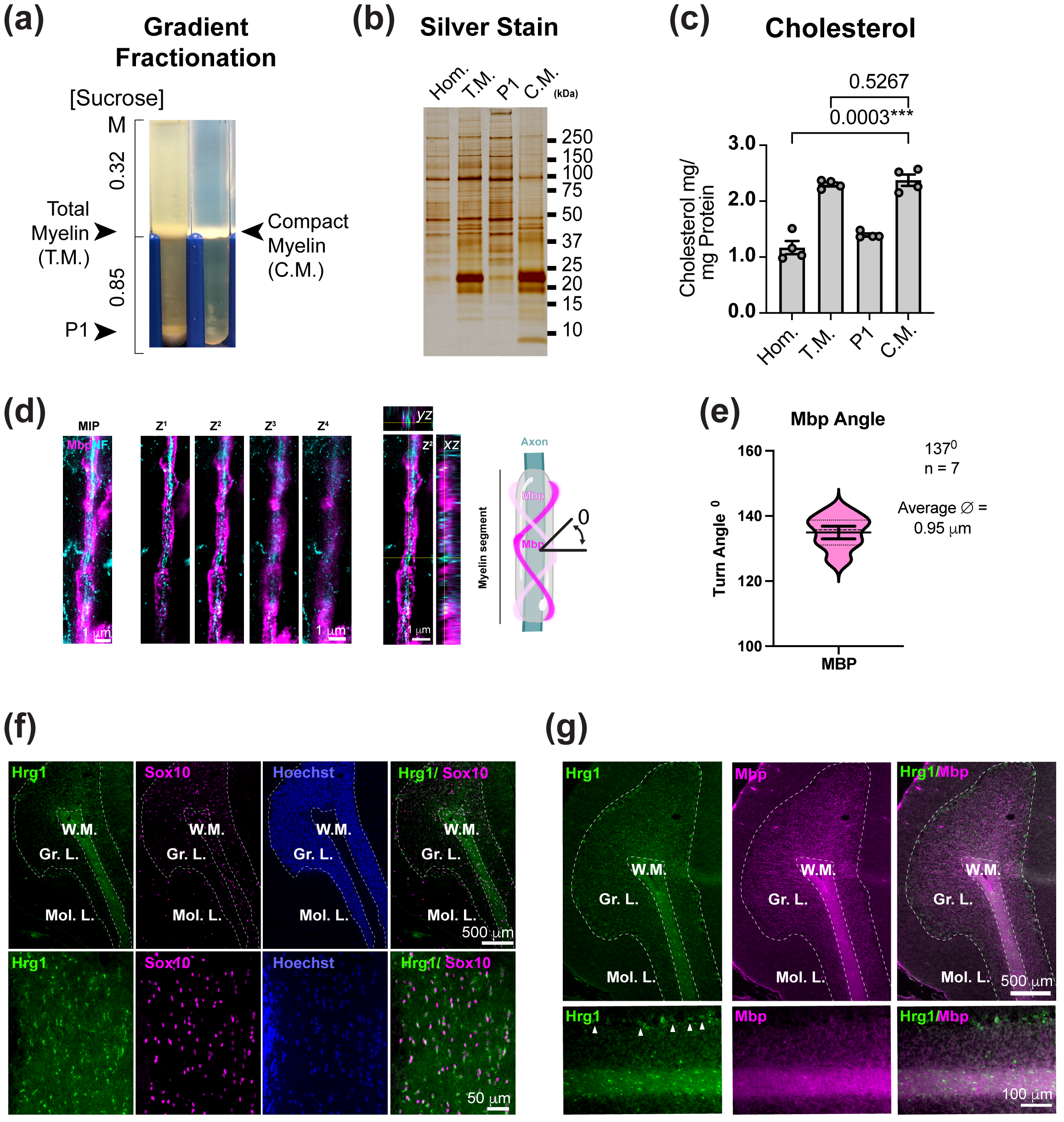

Supplement: Supplementary file 2 — Figure S2. Biochemical and histochemical analysis of CNS myelin. Related to Figures 3 and 4. (a) Typical myelin fractionations generated from sucrose gradient fractionation of rat CNS. The first round of centrifugation reveals a buoyant layer (left centrifuge tube) that is off white yellow in color termed as total myelin (T.M.). After further centrifugation and washing of the total myelin, the buoyant layer is white with a fluffy consistency, which when pelleted and washed is compact myelin (C.M.). (b) 15 ◻g of myelin fractions were separated on Tris‐Tricine gels and stained for total protein by silver method. Note the enrichment of low molecular weight proteins in T.M. and C.M. lanes. (c) Amplex red total cholesterol assay performed on purified lipids from myelin fractions. Cholesterol was measured against a standard curve. Four biological replicates are presented as single points on each histogram. All unpaired t‐tests performed with Welch's correction, values deemed significant as p < .05(*), <.005(**), and <.0005(***), and ns as non‐significant. (d) STED imaging of CNS myelin stained for Mbp (magenta) and neurofilament heavy (NF) (cyan). Left pane is maximum projection and individual z‐stacks through the projection are from left to right. Orthogonal view of a single z plane is shown on the right. Scale bars of 1 μ are shown. Note the helical distribution of Mbp around the axon, which is illustrated. (e) Measurement of angles of Mbp turning relative to axon represented as a violin plot from a minimum of four biological replicates. Angles measured using Fiji and analyzed in GraphPad Prism and data shown as a violin plot with mean and error bars represent S.E.M. (f) Low magnification epifluorescent images for Hrg1 (green), Sox10 (magenta), and nuclei (blue) in cerebellar white matter regions. Scale bars of 500 and 50 μ are shown. (g) Epifluorescent images of Hrg1 (green) and Mbp (magenta) immhunohistochemistry localization in cerebellar white matter regions. Hrg1 i [file GLIA-73-399-s002.tif]

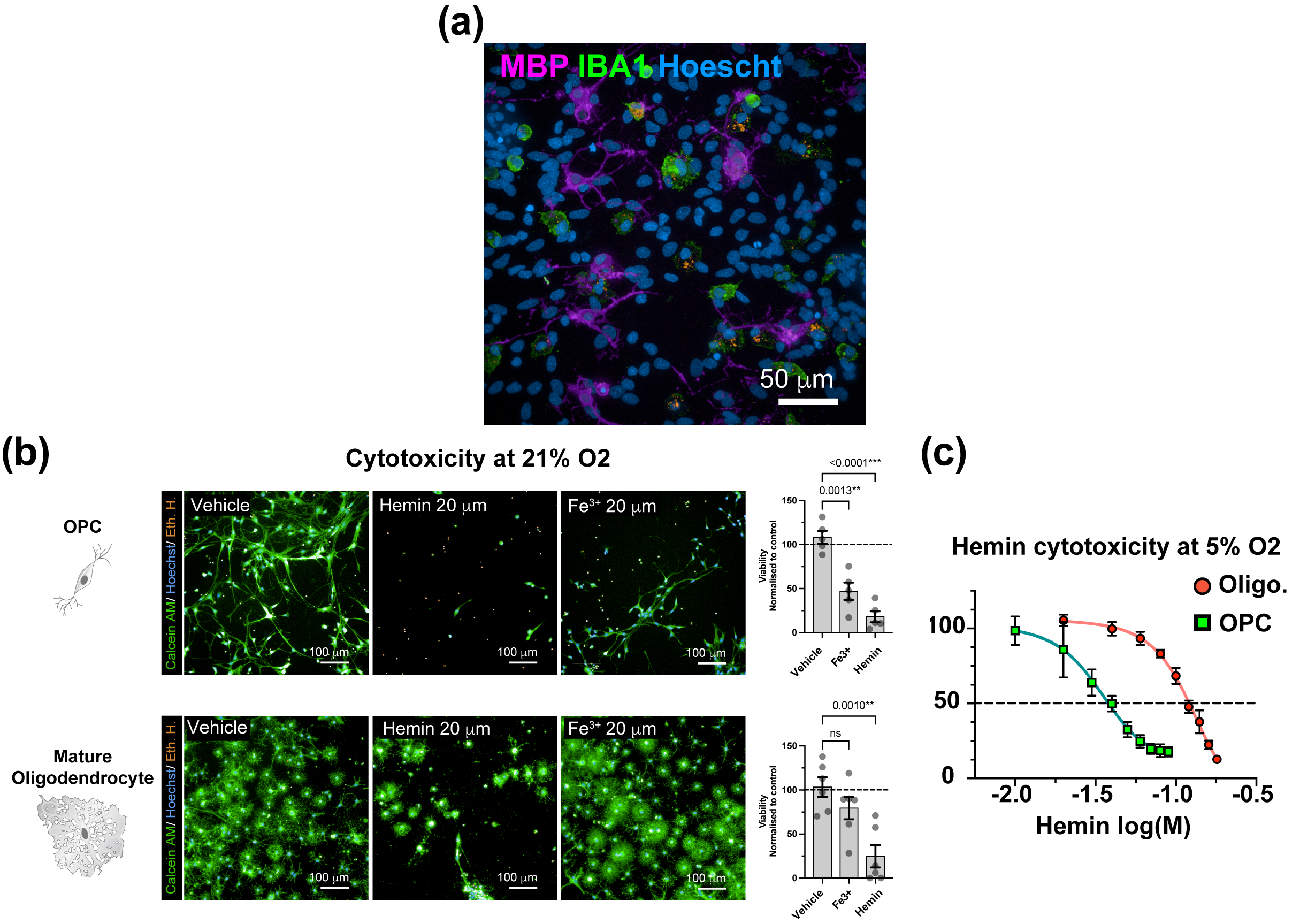

Supplement: Supplementary file 3 — Figure S3. Metalloporphyrin treatment of cell in vitro. Related to Figure 5. (a) Image of oligodendrocytes (Mbp), microglia (Iba1) ZnMP (hot orange) uptake compared to all other cells present detected with Hoechst. Supporting data to Figure 5d–f. Scale bar of 50 μ is shown. (b) Live/Dead assay of primary rat oligodendrocyte precursor cells (OPCs) top panel and primary rat oligodendrocytes (bottom panel) cultured at 21% atmospheric oxygen and treated with 20 ◻M hemin or 20 ◻M iron as found in ferric citrate for 24 h. Live cells contain Calcein AM dye and dead cells are ethidium homodimer (Eth. H.) positive. Cells were imaged and quantified using a semi‐automated pipeline in Harmony software. Data are presented as histograms showing mean ± S.E.M. from an 5 to 6 biological replicates and scale bars of 100 μ are shown. (c) MTT viability assay of OPCs (green) and oligodendrocytes (red) cultured at 5% oxygen and treated with increasing concentrations of hemin (oxidized heme). EC50 values were calculated using GraphPad prism v 9 from four biological replicates. Note the increased in EC50 values for oligodendrocytes compared to OPCs. [file GLIA-73-399-s004.tif]

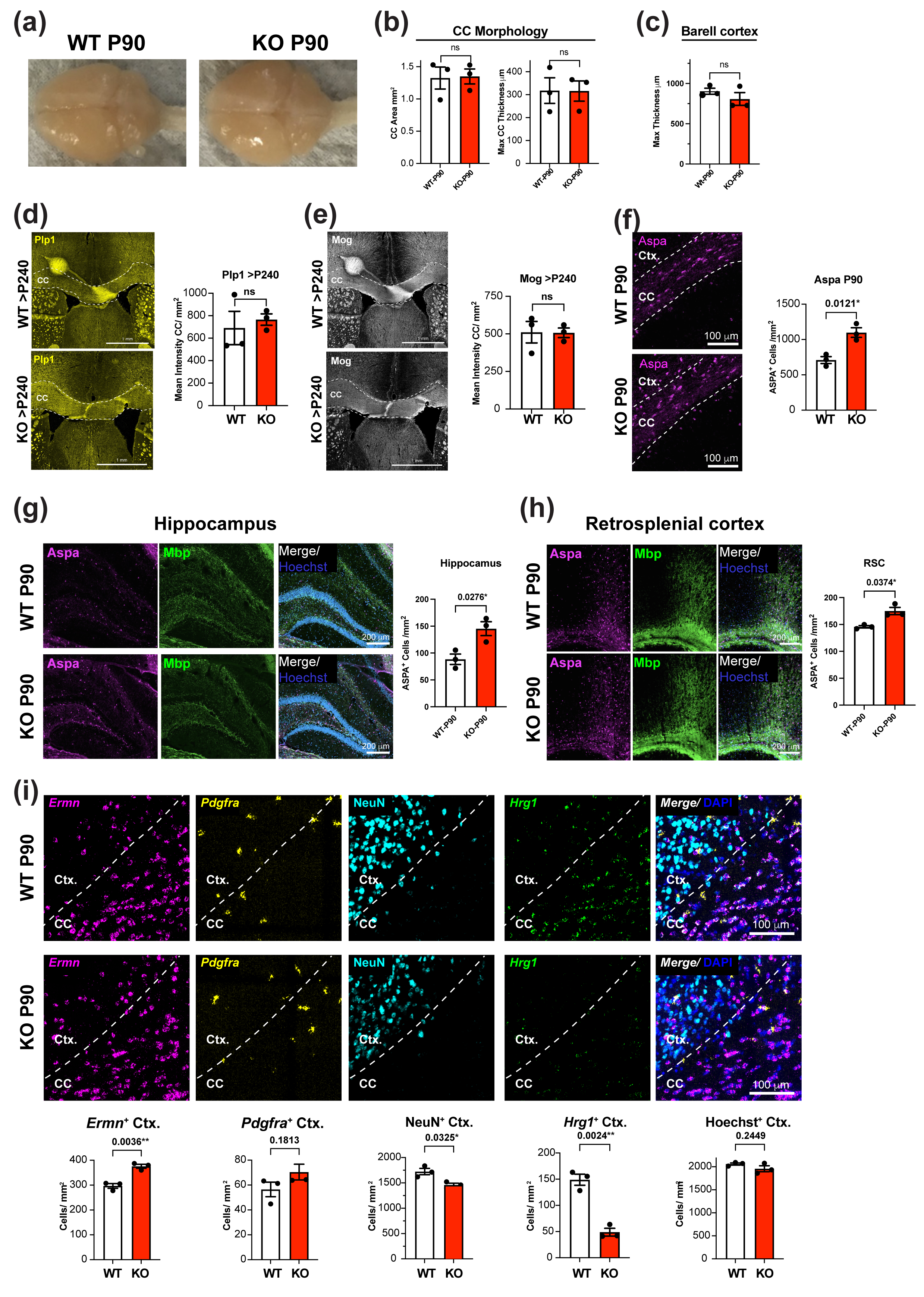

Supplement: Supplementary file 4 — Figure S4. Morphological and cellular assessment in Hrg1 deficient animals. Related to Figure 7. (a) Fixed mouse brains from Hrg1 deficient and littermate controls from P90 animals. (b) Histograms of manual analysis of corpus callosum (CC) morphology for gross area and maximum thickness in Hrg1 deficient and littermate controls from P90 animals. (c) Histograms of manual analysis of cortical thickness in the mouse barrel cortex in Hrg1 deficient and littermate controls from P90 animals. (d) Representative stitched confocal images from mouse coronal sections stained for Plp1 (Yellow) in Hrg1 deficient mice (KO) and wild type (WT) littermates at >P240 (8 months of age or older) and quantified for Plp1 levels in the CC. Data presented as mean ± S.E.M. of 3 biological replicates. Scale bar of 1 mm is shown. Note the levels of Plp1 are not significantly different between Hrg1 deficient mice and controls. (e) Representative stitched confocal images from mouse coronal sections stained for Mog (Gray scale) in Hrg1 deficient mice (KO) and wild type (WT) littermates at >P240 (8 months of age or older) and quantified for Mog levels in the CC. Data presented as mean ± S.E.M. of 3 biological replicates. Scale bar of 1 mm is shown. Note the levels of Mog are not significantly different between Hrg1 deficient mice and controls. (f) Maximum projection of confocal image from the mouse CC in Hrg1 deficient mice (KO) and wild type (WT) littermates at P90 (3 months old) for the mature oligodendroglial marker Aspartoacylase (Aspa) shown as magenta. Cells were quantified manually and normalized to the area per mm2 and data presented as mean ± S.E.M. of 3 biological replicates. Scale bar of 100 μ is shown. (g and h) Immunohistochemistry for Apsa (magenta), Mbp (green) and nuclei (blue) in the hippocampus (g) and retrosplenial cortex (h) from Hrg1 deficient and littermate controls at age P90. Manual quantification of Aspa cell numbers are shown in their respective histograms. Scale bars of [file GLIA-73-399-s006.tif]

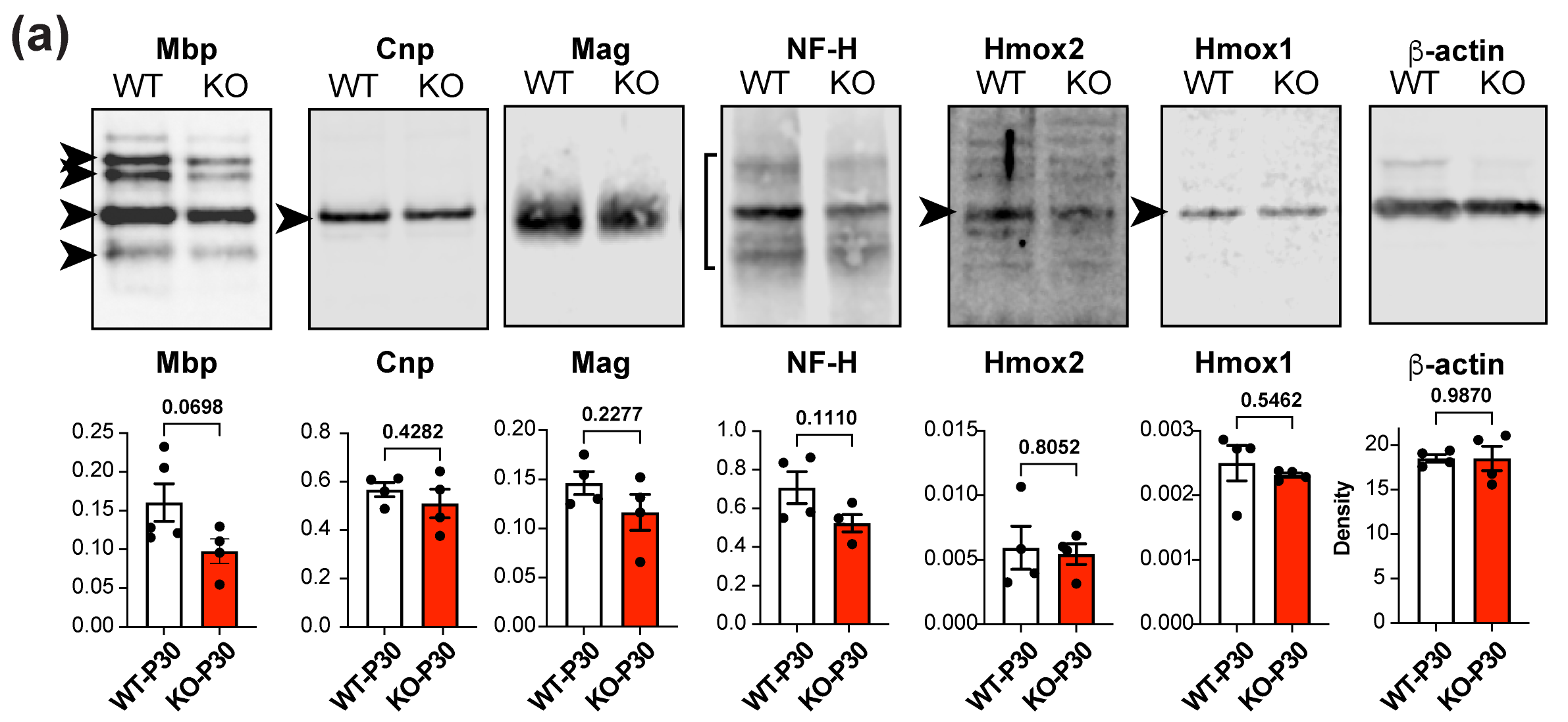

Supplement: Supplementary file 5 — Figure S5. Myelin protein levels in Adult Hrg1 mutant. Related to Figure 7. (a) Representative western blots of whole CNS homogenates from P30 (1 month old) Hrg1 deficient mice (KO) and wild type (WT) littermates for myelin proteins Mbp, Cnp, and Mag, neurofilament‐heavy (NF‐H), heme catabolism enzymes (Hmox2 and Hmox1), and β‐actin as a loading control. Densitometric analyses of westerns normalized to respective β‐actin levels are shown below, representing means ± S.E.Ms. Western blot fluorescent images are depicted in gray scale and biological replicates of 4–5 are presented as single points on each histogram. All blots were repeated at least two times. Arrowheads and square bracket indicate the bands used for western blot quantifications. [file GLIA-73-399-s003.tif]
