# Supplementary material for: Oligodendrocyte Slc48a1 (Hrg1) encodes a functional heme transporter required for myelin integrity
Source: Glia. 2024 Nov 6;73(2):399–421. doi: 10.1002/glia.24641 (PMC11662986; doi:10.1002/glia.24641)
Supplement: Supplementary file 6 — Table S1. Supporting Information. [file GLIA-73-399-s005.pdf]

Table S1

REAGENT or RESOURCE	SOURCE	IDENTIFIER
Antibodies		
Rat anti-MBP (aa82-87)	Bio-Rad	Cat# MCA409S; RRID:AB_325004
Chicken anti-Neun	Merck	Cat# ABN91; RRID:AB_11205760
Mouse anti-MOG	Merck	Cat# MAB5680; RRID:AB_1587278
Rabbit anti-Neurofilament 200 (NF-H)	Merck	Cat# N4142; RRID:AB_477272
Mouse anti-Synaptophysin	Merck	Cat# S5768; RRID:AB_477523
Rabbit Anti-Caspr1	Abcam	Cat# ab34151; RRID:AB_869934
Rabbit anti-Hrg1 (Western blotting; 1: 500 dilution)	Iqbal Hamza, UMD	Pek et al.
Rabbit anti-Hrg1 (Immunofluorescence; 1: 100 dilution)	Rosemary O'Connor, UCC	O'Callaghan et al.
Mouse anti-Hrg1 (Immunofluorescence; 1: 100 dilution)	Rosemary O'Connor, UCC	O'Callaghan et al.
Rabbit anti-HMOX2	CST	Cat# 32790S; RRID:AB_2799030
Rabbit anti-FTH1	CST	Cat# 3998S; RRID:AB_1903974
Mouse anti-Cytochrome C (Cycs)	Abcam	Cat# ab110325; RRID:AB_10864775
Rabbit anti-TOMM20	Abcam	Cat# ab186735; RRID:AB_2889972
Mouse Anti-Mitochondria	Abcam	Cat# ab92824; RRID:AB_10562769
Goat anti-SOX10	Biotechne	Cat# AF2864; RRID:AB_442208
Mouse anti-CNPase (Cnp)	Merck	Cat# C5922; RRID:AB_476854
Mouse anti MAG, clone 513	Merck	Cat# MAB1567; RRID:AB_11214010
Mouse anti-heme oxygenase 1 (Hmox1)	Abcam	Cat# ab13248; RRID:AB_2118663
Mouse anti-β-Actin	Merck	Cat# A1978; RRID:AB_476692
Rabbit anti-myelin Plp	Abcam	Cat# ab28486; RRID:AB_776593
Rabbit anti-ASPA	Abcam	Cat# ab239522; RRID N/A
Rabbit anti-Iba1	Alpha Laboratories	Cat # 019-19741; RRID:AB_839504
Goat anti-Olig2	Biotechne	Cat# AF2418; RRID:AB_2157554

Donkey anti-Goat IgG (H+L) Cross-Adsorbed Secondary Antibody, Alexa Fluor 488	Thermo Fisher Scientific	Cat# A11055; RRID:AB_2534102
Donkey anti Goat IgG (H+L) Secondary Antibody, Alexa Fluor 647, Invitrogen	Thermo Fisher Scientific	Cat# A21447; RRID:AB_2535864
Donkey anti-Rabbit IgG (H+L) Highly Cross-Adsorbed Secondary Antibody, Alexa Fluor 488	Thermo Fisher Scientific	Cat# A21206; RRID:AB_2535792
Donkey anti-Rabbit IgG (H+L) Highly Cross-Adsorbed Secondary Antibody, Alexa Fluor 555	Thermo Fisher Scientific	Cat# A31572; RRID:AB_162543
Donkey anti Rabbit IgG (H+L) Highly Cross Adsorbed Secondary Antibody, Alexa Fluor 647	Thermo Fisher Scientific	Cat# A31573; RRID:AB_2536183
Donkey anti Rat IgG (H+L) Highly Cross Adsorbed Secondary Antibody, Alexa Fluor 488	Thermo Fisher Scientific	Cat# A21208; RRID:AB_2535794
Donkey anti-Rat IgG (H+L) Highly Cross-Adsorbed Secondary Antibody, Alexa Fluor Plus 647	Thermo Fisher Scientific	Cat# A48272; RRID:AB_2893138
Donkey anti-Mouse IgG (H+L) Highly Cross-Adsorbed Secondary Antibody, Alexa Fluor 488	Thermo Fisher Scientific	Cat A21202; RRID:AB_141607
Donkey anti Mouse IgG (H+L) Secondary Antibody, Alexa Fluor 555, Invitrogen	Thermo Fisher Scientific	Cat# A31570; RRID:AB_2536180
Goat Anti-Rat IgG (IRDye® 800CW)	LI-COR Biosciences	Cat# 925-32219; RRID:AB_2721932
Goat Anti-Mouse IgG (IRDye 800CW)	LI-COR Biosciences	Cat# 925-32210; RRID:AB_2687825
Goat Anti-Rabbit IgG (IRDye 800CW)	LI-COR Biosciences	Cat# 925-32211; RRID:AB_2651127
Donkey Anti-Chicken IgG (IRDye® 680RD)	LI-COR Biosciences	Cat# 925-68075; RRID:AB_2814924
Goat Anti-Rat IgG (IRDye 680RD)	LI-COR Biosciences	Cat# 925-68076; RRID:AB_2814913
Goat Anti-Mouse IgG (IRDye 680RD)	LI-COR Biosciences	Cat# 925-68070; RRID:AB_2651128
Goat Anti-Rabbit IgG (IRDye 680RD)	LI-COR Biosciences	Cat# 925-68071; RRID:AB_2721181
Anti-A2B5 Antibody, clone A2B5-105	Merck	Cat# MAB312; RRID:AB_11213452
Anti-Mouse IgM MicroBeads	Miltenyi Biotec	Cat# 130-047-301; RRID:AB_244358

Goat anti-rat IgG, abberior STAR 580	abberior Instruments GmbH	Cat# ST580-1007-500UG
Goat anti-rabbit IgG, abberior STARRED	abberior Instruments GmbH	Cat# STRED-1002-500UG
Bacterial and virus strains		
N/A		
Biological samples		
Primary rat oligodendrocyte cultures	This paper	
Primary mouse mixed glial cultures	This paper	
Chemicals, peptides, and recombinant proteins		
Hibernate-A	Thermo Fisher Scientific	Cat# A12475-01
HBSS	Thermo Fisher Scientific	Cat# 14170-112
Papain	Worthington Labs	Cat# L5003126
DNAse Type I	Merck	Cat# D5025
Percoll Plus	Cytiva	Cat# 17544501
Red Blood Cell Lysis Buffer	Merck	Cat# R7757
B27 50X Supplement	Thermo Fisher Scientific	Cat# 17504-044
Apo-transferrin	Merck	Cat# T1147
DMEM/F12	Thermo Fisher Scientific	Cat# 11039-021
PDGF (Recombinant Human PDGF-AA)	Peprotech	Cat# 100-13A-100
FGF (Recombinant Human FGF-basic (154 a.a.)	Peprotech	Cat# 100-18B-500
HB-EGF (Recombinant Human HB-EGF)	Peprotech	Cat# 100-47
Insulin Solution	Merck	Cat# I9278
Ferriprotoporphyrin IX chloride (Hemin)	Merck	Cat# H9039-1G

Zn(II) Mesoporphyrin IX (ZnMP)	Santa Cruz Biotechnology	Cat# sc-396862
Tin protoporphyrin IX dichloride (SnPP)	Tocris	Cat# 0747/10
Deferoxamine (DFO)	Merck	Cat# D9533-1G
Hoechst 33342 solution	Thermo Fisher Scientific	Cat# H3570
Tri Reagent	Thermo Fisher Scientific	Cat# AM9738
Direct-zol RNA MicroPrep	Cambridge Bioscience	Cat# R2062
Critical commercial assays		
Live/dead™ Viability/Cytotoxicity Kit, for mammalian cells	Thermo Fisher Scientific	Cat# L3224
MTT Assay Kit (Cell Proliferation) (ab211091)	Abcam	Cat# ab211091
Amplex™ Red Cholesterol Assay Kit	Thermo Fisher Scientific	Cat# A12216
Pierce™ Silver Stain Kit	Thermo Fisher Scientific	Cat# 24612
Qubit Protein Assay Kit	Thermo Fisher Scientific	Cat# Q33212
Deposited data		
N/A		
Experimental models: Cell lines		
N/A		
Experimental models: Organisms/strains		
Rat: Wister/Han	Charles River UK	N/A
Mice: C57	Charles River UK	N/A
Slc48a1 -/- 2 base pair deletion in background strain of SVJ129/C57BL6J	Iqbal Hamza, UMD	Pek et al.[19]
Oligonucleotides		

β-actin<br><br>forward 5' AGATGACCCAGATCATGTTTGAGA 3', reverse 5' ACCAGAGGCATACAGGGACA 3'	This paper	N/A
Slc48a1 (hrg1)<br><br>forward 5' TTCGTCTGGACGGTGGTCTA 3'<br><br>reverse 5' GTGAGTCACCAAGACCCACA 3'	This paper	N/A
Mfsd7b<br><br>forward 5' GTTCTTCGAGACAGCCCCC 3'<br><br>reverse5' AGAACAAAGGGGACGTTCCTG 3'	This paper	N/A
Tfrc<br><br>forward 5' CCGTTGTTGAGGCAGACCTT 3', reverse 5' CCAGATGACTGAGATGGCGG 3'	This paper	N/A
Hmox1<br><br>forward 5' TTAAGCTGGTGATGGCCTCC 3', reverse 5' GTGGGGCATAGACTGGGTTC 3'	This paper	N/A
Bach1<br><br>forward 5' AGCTCGACTGCATCCAGAAC 3', reverse 5' GTTCTGCTTTGTCTCCCCGA 3'	This paper	N/A
MBP<br><br>forward 5' GCCTGTCCCTCAGCAGATTT 3', reverse 5' GTCGTAGGCCCCCTTGAATC 3'	This paper	N/A
PLP<br><br>forward 5' GGCGACTACAAGACCACCAT 3', reverse 5' AATGACACACCCGCTCCAAA 3'	This paper	N/A
MAG<br><br>forward 5' GGCGCTTCTCACTCTCATACT 3', reverse 5' CGGGAGACAACCCTCATGTC 3'	This paper	N/A
Recombinant DNA		
N/A		
Software and algorithms		
MyelTracer v1.3.1	Kaiser et al.[69]	https://github.com/HarrisonAllen/MyelTracer

GraphPad Prism version 9.0.0 for Mac	GraphPad Software/ Dotmatics, USA	https://www.graphpad.com/features
OMERO.web 5.22.1	University of Dundee, UK	https://www.openmicroscopy.org/omero/
Other		
Operetta CLS	Perkin Elmer	https://www.perkinelmer.com/category/operetta-cls-high-content-analysis-system
Leica Bond RX	Leica Microsystems	https://www.leicabiosystems.com/ihc-ish/ihc-ish-instruments/bond-rx/
RNAscope™ 2.5 LS Probe- Mm-Slc48a1-C2	Biotechne	Cat# 585048-C2
RNAscope™ 2.5 LS Probe- Mm-Syt1	Biotechne	Cat# 491838
RNAscope™ 2.5 LS Probe- Mm-Ermn-C4	Biotechne	Cat# 527378-C4
RNAscope™ 2.5 LS Probe- Mm-Pdgfra	Biotechne	Cat# 480668
70 micron strainer	Greiner	Cat# 542070
LS MACS Columns	Miltenyi Biotec	Cat# 130-042-401
